# New Analytical Method for Quantifying Flavoring Chemicals of Potential Respiratory Health Risk Concerns in e-Cigarette Liquids

**DOI:** 10.3389/fchem.2021.763940

**Published:** 2021-10-28

**Authors:** Michelle K. Page, Maciej L. Goniewicz

**Affiliations:** Department of Health Behavior, Roswell Park Comprehensive Cancer Center, Buffalo, NY, United States

**Keywords:** flavors, flavorings, flavoring chemicals, electronic cigarettes, e-cigarettes, E-liquids, E-cigarette refill solutions, vaping (Min 5-Max 8)

## Abstract

Numerous flavoring chemicals are added to e-cigarette liquids to create various flavors. Flavorings provide sensory experience to users and increase product appeal; however, concerns have been raised about their potential inhalation toxicity. Estimating potential health risk of inhaling these chemicals has been challenging since little is known about their actual concentrations in e-cigarette products. To date, a limited number of analytical methods exist to measure the concentrations of flavoring chemicals in e-cigarette products. We have developed an analytical method that accurately and precisely measures the concentrations of 20 flavoring chemicals of potential inhalation risk concerns: 2,3,5-trimethylpyrazine, acetoin, benzaldehyde, benzyl alcohol, butanoic acid, dl-limonene, ethyl maltol, ethyl salicylate, ethyl vanillin, eucalyptol, eugenol, furaneol, isovanillin, l-menthol, maltol, methyl salicylate, pulegone, *trans*-cinnamaldehyde, triacetin, and vanillin. Calibration and QC solutions were prepared in 50:50 propylene glycol (PG):vegetable glycerin (VG) and 5% H_2_O and flavoring concentrations ranging from 0.02 to 10.00 mg/ml. Samples of commercial e-cigarette liquids, calibration and QC solutions were combined with 30 µL of an internal standard mix (benzene-d6, pyridine-d5, chlorobenzene-d5, naphthalene-d8 and acenaphthene-d10; 1 mg/ml each) and were diluted 100-fold into methanol. Analysis was performed on an Agilent 7890B/7250 GC/Q-TOF using a DB-624UI column (30 m x 0.25 mmID x 1.4 μm film thickness), with a total runtime of 13.5 min. Calibration curves were fit using a weighted quadratic model and correlations of determination (*r* ^2^) values exceeded 0.990 for all chemicals. Bias and precision tests yielded values less than 20% and lower limits of quantitation (LLOQ) ranged from 0.02 to 0.63 mg/ml. Over 200 commercially available products, purchased or collected from adult e-cigarette users and spanning a range of flavor categories, were evaluated with this method. Concentrations of pulegone, a suspected carcinogen, varied from below limit of quantitation (BLOQ) to 0.32 mg/ml, while acetoin and vanillin, known precursors to more cytotoxic byproducts, ranged from BLOQ to 1.52 mg/ml and from BLOQ to 16.22 mg/ml, respectively. This method features a wide dynamic working range and allows for a rapid routine analysis of flavoring additives in commercial e-cigarette liquids.

## Introduction

Flavoring chemicals are a main constituent of e-cigarette liquids and help impart either a characteristic flavor or contribute to the overall sensory experience of e-cigarette users (vapers). Over 7,000 e-cigarette liquid flavors are available to consumers (Zhu et al., 2014), with unlimited variations of added flavoring chemicals and their concentrations. The accessibility of flavors has led to higher likability of e-cigarette products (Kim et al., 2016) and higher initiation rates of vaping (Leventhal et al., 2019). As a result, an increase in preference and usage of flavored e-cigarettes has been shown among multiple population groups (Harrell et al., 2017), observed most strikingly among the youth population (Ambrose et al., 2015; Harrell et al., 2017). Users more frequently site flavors as their reason for initiation and usage (Pepper et al., 2016; Harrell et al., 2017; Russell et al., 2018) and the exposure from increased consumption of flavored liquids and their chemical flavorings, is worrisome from a public health perspective.

Previous qualitative methods have established the identities of the flavoring chemicals commonly used in tobacco products (Krüsemann et al., 2018), including e-cigarette liquids (Hutzler et al., 2014; Czoli et al., 2019). Among the most frequently reported flavorings in e-cigarette liquids, aldehydes (ethyl vanillin, vanillin, benzaldehyde and cinnamaldehyde), alcohols (l-menthol, benzyl alcohol and furaneol), esters (triacetin), ketones (ethyl maltol, maltol, acetoin) and terpenes (limonene, pulegone) are the most common (Hutzler et al., 2014; Tierney et al., 2016; Behar et al., 2018; Omaiye et al., 2019b; Czoli et al., 2019; Hua et al., 2019; Krüsemann et al., 2021). While considered “generally recognized as safe” (GRAS) for consumption, concerns have been raised about the potential inhalation toxicity associated with these chemicals. Initial findings suggest a link between inhalation toxicity of e-cigarettes and flavorings (Bahl et al., 2012; Leigh et al., 2016), while more recent *in vitro* studies have demonstrated specific chemicals such as cinnamaldehyde, benzaldehyde, ethyl vanillin, ethyl maltol and vanillin to be highly cytotoxic to respiratory cells (Behar et al., 2018; Hua et al., 2019; Rickard et al., 2021), as well as disruptive to normal cellular and immune function (Gerloff et al., 2017; Hickman et al., 2019). Selected flavoring chemicals have been also shown to form free radicals when heated in e-cigarette devices (Muthumalage et al., 2017; Bitzer et al., 2018), as well as known carcinogens such as benzene (Pankow et al., 2017), while others react with solvents used in e-cigarette liquids to form more highly cytotoxic acetal byproducts (Erythropel et al., 2019; Jabba et al., 2020). Further, ethyl vanillin and vanillin have been revealed to contain potentially addictive properties (Truman et al., 2019).

While exposure to flavoring chemical classes such as aldehydes and alcohols are more widely studied, the use of additional chemicals in e-cigarette liquids are also concerning. For example, pulegone is a suspected carcinogen at high concentrations and has subsequently been banned from food products in 2018 (Kidwell, 2018). Its presence in e-cigarette liquids; however, remains unregulated as the margin of exposure in some marketed e-cigarette liquids have been shown to far exceed that found in food (Jabba and Jordt, 2019). Further, acetoin is a known precursor to diacetyl formation in e-cigarette liquids (Vas et al., 2019), where diacetyl has been identified in a large number of sweet flavored liquids (Farsalinos et al., 2015). Importantly, occupational inhalation of diacetyl has previously demonstrated to cause serious human respiratory outcomes (bronchiolitis obliterans) (Kreiss et al., 2002). Similarly, the addition of triacetin to e-cigarette liquids has been correlated with increases in harmful smoke constituents, such as formaldehyde hemiacetals, acrolein and acetaldehyde (Vreeke et al., 2018), owing to the degradation of triacetin at high temperatures (Laino et al., 2012).

As research continues to focus on the health effects of flavoring chemicals used in flavored e-cigarette liquids, accurately estimating potential toxicity has been challenging since concentrations of these chemicals are generally not known. E-cigarette liquid manufacturers are not required to report chemical constituents or concentrations. Several studies have published concentrations of common flavoring chemical additives; however, validation of the methods used to determine accuracy of the reported results are limited. This study aimed to develop an accurate and highly efficient method, spanning a wide concentration range for the following 20 chemicals commonly found in e-cigarette liquids that are of potential inhalation concern: 2,3,5-trimethylpyrazine, acetoin, benzaldehyde, benzyl alcohol, butanoic acid, dl-limonene, ethyl maltol, ethyl salicylate, ethyl vanillin, eucalyptol, eugenol, furaneol, isovanillin, l-menthol, maltol, methyl salicylate, pulegone, *trans*-cinnamaldehyde, triacetin and vanillin. Using this validated method, over 200 commercial e-cigarette liquids were assessed for concentrations of these 20 chemicals.

## Materials and Methods

### Chemicals

Neat standards for the 20 flavoring chemicals and internal standards were purchased from Acros Organics (Fair Lawn, NJ), Alfa Aesar (Haverhill, MA), Cambridge Isotopes (Tewksbury, MA), Santa Cruz Biotechnology (Dallas, TX), Sigma Aldrich (St. Louis, MO), and TCI (Portland, OR) (Supplementary Table S1). The solvents methanol (LCMS-grade) and water (HPLC-grade) were obtained from Fisher Chemical (Waltham, MA), propylene glycol (PG) from Acros Organics and vegetable glycerin (VG) from Alfa Aesar.

### Preparation of Working Solutions

A solvent solution of 50:50 (%:%) propylene glycol (PG) and vegetable glycerin (VG) was first prepared by combining 475 ml of each along with 50 ml of HPLC-grade water and mixing for 15 min using a magnetic stir plate (Fisher Scientific Isotemp™, Waltham, MA) at 350 rpm and ambient temperature. The solution settled for 0.5 h to allow removal of trapped air bubbles from the mixing process. This solution was utilized for subsequent preparation of calibration, quality control and fortified matrix samples used in the validation of this method. Storage of the solution was in ambient dark conditions and prepared as needed.

A 20 mg/ml working solution containing the follow chemicals was prepared by weighing 2.0000 ± 0.0005 g of each solid neat standard using a precision balance (0.008–220 g, Mettler-Toledo, Columbus, OH) and dissolving into methanol: acetoin, ethyl maltol, ethyl vanillin, furaneol, isovanillin, maltol, l-menthol and vanillin. L-menthol was first crushed to a fine powder using a ceramic mortar and pestle. The mixture was hand-vortexed at 3,200 rpm using a vortex mixer (Fisher Scientific) for a minimum of 5 min or until all visible granules were dissolved. The working solution was stored in dark, 4°C conditions and prepared monthly.

A 1 mg/ml working internal standard (IS) solution was prepared into methanol, where 100 μL of benzene-d6, chlorobenzene-d5 and pyridine-d5 and 100.0 ± 0.05 mg of naphthalene-d8 and acenapthene-d10 were measured and the solution hand-vortexed for a minimum of 5 min. Internal standard solution was kept in ambient dark conditions and prepared yearly.

### Preparation of Working Calibration and Quality Control Standards

Ten calibration and nine quality control (QC) concentrations were prepared ranging from 0.02 to 10.00 mg/ml and 0.03–8.00 mg/ml, respectively by serial dilutions (2-fold) starting with the most concentrated level (Supplementary Table S2). Here, 100 and 80 μL of each liquid neat standard (Supplementary Table S1) and 4 ml of the 20 mg/ml working solution were gently mixed with 3.7 and 4.96 ml of 50:50 PG:VG solution, respectively for 0.5 h using a vertical multi-function rotator (Grant Instruments, Shepreth, United Kingdom). Given the high concentration of the flavoring chemicals in the calibration standards as well as high concentrations expected in e-cigarette liquids, detector saturation with direct injection was of concern. To reduce this effect, each standard was diluted 100-fold prior to injection using similar methodology to dilute-and-shoot LCMS (Greer et al., 2021), by adding 30 μL of each to 30 μL of internal standard solution and 3 ml of methanol. Calibration and QC standards were stored in 4 °C dark conditions and prepared monthly.

### Preparation of Fortified Matrix Samples for Method Validation

Fortified matrix samples to validate the bias and precision of the method were prepared in triplicate at the following three concentrations within the instrument linear range: 1. approximately 3 times the lowest level, 2. middle of the range 3. within at least 70% of the highest level. Given the complexity of the ranges, this required the preparation of six fortified samples (at concentrations of 0.04, 0.10, 0.88, 1.75, 3.50 and 7.00 mg/ml). The most concentrated fortified sample was prepared first by adding each neat standard (70 μL) and 20 mg/ml working standard (3.5 ml) into the PG:VG solution (5.59 ml) and gently mixing for 0.5 h, followed by subsequent serial dilutions. To assess the lower limit of quantitation (LLOQ) for each chemical, additional fortified matrix samples were prepared at concentrations 0.5–2 times the lowest level in the instrument linear range. This required the preparation of five samples (at concentrations of 0.01, 0.04, 0.07, 0.10 and 0.27 mg/ml) and were prepared from individual dilutions of a working intermediate (1 mg/ml). Dilution capability of the method was evaluated by preparing independent fortified matrix samples at concentrations of 5 and 10 mg/ml, using previously described procedures, and 18.6 mg/ml, where solid and liquid neat standards were dissolved directly into 50:50 PG:VG. Similar to calibration and QC standards, all fortified matrix samples were diluted 100-fold with methanol and internal standard solution prior to analysis.

### Selection of Commercial E-Cigarette Liquids

To test the capacity of the method, previously obtained e-cigarette liquids were selected for analysis based on the availability of popular flavors. Roughly half of the liquids were either purchased online or in vape shops (53%), while the remainder (47%) were collected from participants from observational studies of adult e-cigarette users. Most liquids were from the US (90%), with several from Australia (7%), the United Kingdom (3%) and one liquid from Canada. This included 215 in total and incorporated 13 of 16 flavor categories from a recently published e-cigarette liquid flavor wheel (Krüsemann et al., 2019), increasing the probability of detecting the targeted flavorings chemicals of this method. Such flavor categories from the flavor wheel included Fruit (further delineated as tropical, berry, citrus and other), Dessert, Candy, and Menthol/Mint, in addition to Tobacco. E-cigarette liquids were stored in 4 °C dark conditions prior to analysis and were brought to room temperature and mixed for 1 hour using a vertical multi-function rotator. As with the calibration standards and fortified matrix samples, e-cigarette liquids were diluted 100-fold with methanol and internal standard solution prior to analysis.

### Instrumental Analysis

Sample analysis was performed on a 7890B/7250 GC/Q-TOF (Agilent Technologies, Santa Clara, CA), equipped with a PAL RSI 120 autosampler (CTC Analytics, Zwingen, Switzerland). Separation of chemicals was achieved using an Agilent DB-624UI (30 m x 0.25 mmID x 1.4 μm film thickness) column. To ensure adequate settling of the stationary phase between injections and more reproducible retention times of early eluting chemicals, the column equilibrated for 2 min at initial conditions between injections. After pre-rinsing the needle with methanol, 1 μL of the prepared aliquot was injected into 320°C with a split of 20:1. Initial oven conditions started at 60°C and were held for 1 min. The temperature was then increased at a rate of 30°C/min until reaching 225°C and held for an additional 4 min. Post-acquisition, the oven was ramped to 280°C and held for 1 min to help clean residual carryover. Total analysis time was 13.5 min. Elution from the column into the mass spectrometer occurred at 250°C and source and quadrupole temperatures were held at 230°C and 150°C, respectively. Positive ionization was performed using low-EI (15 eV) with emission of 0.2 μA. Q/TOF scan range was between 50 and 250 amu, with acquisition rate and time of five spectra/sec and 200 ms/spectrum, respectively. Carrier flow (helium) was held constant at 2.0 ml/min, while quench (helium) and collision (nitrogen) gases were held at 2.0 ml/min and 1.5 ml/min, respectively. Needle rinses post-injection were first in acetone and followed by methanol.

Each acquisition batch consisted of up to 75 e-cigarette liquids, as well as one complete set of calibration (0.02–10.00 mg/ml) and QC (0.03–8.00 mg/ml) standards injected prior to the e-cigarette liquids and one complete set injected after. Using the responses from both sets of calibration standards, a calibration curve was plotted to measure the concentrations of chemicals identified in the e-cigarette liquids in the batch. Data review was performed using Agilent MassHunter software (Quantitation, v10.2) and automated method processing. Computer generated peak assignments and integrations were reviewed and corrected when applicable. Calibration curves were plotted for each chemical, using peak area and the internal standard method. Commercial liquids with chemicals exceeding the upper quantitation limit of the calibration curve range were diluted to a concentration near the middle of the calibration range (1 mg/ml). Dilutions of 2, 5 and 10X were performed with reduction of initial e-cigarette liquid volume, while dilutions of 20 and 50X also required adjusted final volumes of the solvent. To account for differing final volumes, the volume of the internal standard solution added was adjusted likewise to allow recovery within ±20% compared to the calibration. Diluted liquids were re-injected with corresponding calibrators and QC.

## Method Validation

Validation of this method followed recommendations from the Scientific Working Group for Forensic Toxicology (SWGTOX) (Scientific Working Group for Forensic, 2013). To assess the suitability of internal standard concentration and assignments, relative response factors (RRF) across calibration standards for each chemical were calculated using the following equation:

$$RRF=\frac{A_{s}\;x\;C_{\mathrm{is}}}{A_{\mathrm{is}}\;x\;C_{s}}$$

Where:

A_s_ = area response of the chemical.

A_is_ = area response of the internal standard.

C_s_ = concentration of the chemical.

C_is_ = concentration of the internal standard.

Relative standard error (RSE) was calculated to determine the acceptability of the curve models, using the following equation:

$$\%\;RSE=100\;\times \;\sqrt{{\left[\overset{n}{\underset{i=1}{\sum }}[\frac{x_{i}^{\prime }-x_{i}}{x_{i}}\right]}^{2}/\left(n-p\right)}$$

Where:

n = number of calibration points

x_*i*_ = expected concentration of chemical in calibration level *i*

x_*i*_’ = measured concentration of chemical in calibration level *i*

p = number of term in the fitting equation (average = 1,  linear = 2, quadratic = 3).  For all 20 chemicals, p = 3

Fortified matrix samples were analyzed in triplicate per batch, where seven batches in total were injected on separate days. Percent recovery of each calibrator, QC and fortified matrix sample were compared within batch (inter-day) and between batches (intra-day) and coefficient of variation (CV) was used to determine precision and accuracy, where a range of ±20% was considered acceptable (Scientific Working Group for Forensic, 2013). Precision was calculated using the following equation:

$$\%CV=\frac{Average\;Concentration}{Standard\;Deviation}\;X\;100$$

Bias was determined using the following equation:

$$\%Bias=\;\frac{Average\;Concentration-Expected\;Concentration}{Expected\;Concentration}\;X\;100$$

LLOQ values for the working calibration range were primarily determined from the lowest calibration level meeting ±20% recovery and CV, and secondly from the results of the LLOQ and carryover analyses. Carryover was assessed with three 50:50 PG:VG and three methanol blank matrix samples following injection of the highest working standards (8 and 10 mg/ml). Dilution capability was determined by targeting concentrations within the working calibration range with two- and 5-fold dilutions of each the 5, 10 and 18.6 mg/ml fortified samples, while 10- and 50-fold dilutions were performed on the 10 mg/ml and 18.6 ml samples only. Stability was assessed by percent recovery of several calibrators injected after 1 month against a newly prepared calibration.

## Results

### Chemical Identification

**FIGURE 1:**
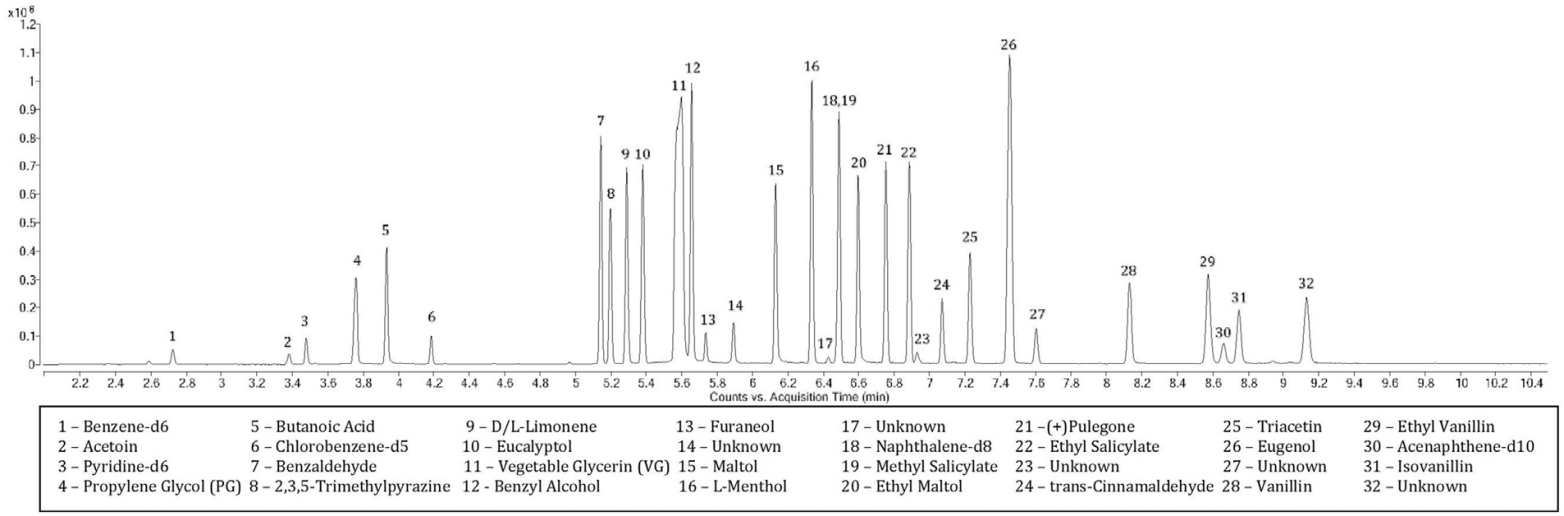
Total Ion Chromatogram of Targeted Flavoring Chemicals and Internal Standards.

Spectral identification and retention times (RTs) were established from independent analysis of each chemical (Table 1). The largest ion was selected for quantitation when possible and secondary ions were chosen at a minimum relative abundance of 10% of the quantitation ion, with exception of benzene-d6 (6.2%). Five chemicals were further assigned tertiary ions meeting the minimum threshold. A m/z window of ±10 ppm was applied to allow slight instrument measurement variations. The earliest eluting peak after internal standard benzene-d6 (2.722 min) was acetoin (3.382 min) while the concluding peak isovanillin eluted at 8.747 min (Figure 1). PG and VG, while not included in the calibration, were observed at RTs of 3.759 and 5.599 min, respectively. Several unknown peaks were observed at RTs of 5.892, 6.431, 6.929, 7.603 and 9.130 min. Tentative identification by comparing spectral breakdown (with the most abundant ions of 121.0654, 123.0810, 121.0655, 147.0810 and 167.0709 m/z, respectively) to the National Institute of Standards and Technology (NIST) library suggest these may be benzaldehyde dimethyl acetal (CAS# 1125–88-8), isopulegone (CAS# 29606–79-9), 2′-hydroxybutyrophenone (CAS# 2887–61-8), cinnamaldehyde dimethyl acetal (CAS# 4364–06-1) and 4-(2-hydroxyethyl)-2,6-dimethoxyphenol (CAS# 20824-4-7), respectively. Identities of these peaks were determined from the analysis of the total ion chromatogram (TIC) rather than through deconvolution processes, which could have revealed additional peaks at the specified retention times. Further, because the NIST was developed using nominal mass and an ionization energy of 70eV, the spectral match to the data produced with high resolution (accurate) mass and low eV may not adequately identify these peaks. Peak resolution, calculated from the difference in retention times of the later and earlier eluting chemicals, divided by the average of the peak widths (Carle, 1972), was greater than 1.5 between each extracted ion current profile (EICP) with exception of ethyl vanillin and naphthalene-d8 (Table 1). Internal standard assignments are listed in Table 1.

**TABLE 1:** Retention time, Resolution, Quantitation and Qualitative Ions and Internal Standard Assignments for Twenty Flavoring Chemicals.

	CAS	Molecular Formula	Molecular Weight (g/mol)	RT (min)	Peak resolution^a^	m/z (±10ppm)					IS Assignment
						1°(Quant)	2°(Qual)	Relative abundance (%)	3°(Qual)	Relative abundance (%)	
Acetoin	513-86-0	C4H8O2	88.11	3.382	4.08	88.0530	73.0295	15.0	---	---	Benzene-d6
Butanoic Acid	107-92-6	C4H8O2	88.11	3.928	10.96	60.0211	73.0293	30.0	---	---	Pyridine-d5
Benzaldehyde	100-52-7	C7H6O	106.12	5.141	2.35	106.0409	77.0395	84.0	105.0335	11.9	Chlorobenzene-d5
2,3,5-Trimethylpyrazine	14667-55-1	C7H10N2	122.17	5.203	3.96	122.0834	81.0583	11.1	---	---	Chlorobenzene-d5
D-Limonene	5989-27-5	C10H16	136.23	5.287	3.91	93.0705	121.1022	28.5	136.1257	33.5	Chlorobenzene-d5
L-Limonene	5989-54-8	C10H16	136.23	5.287	3.91	93.0705	121.1022	28.5	136.1257	33.5	Chlorobenzene-d5
Eucalyptol	470-82-6	C10H18O	154.25	5.381	5.73	139.1129	154.1363	90.2	---	---	Chlorobenzene-d5
Benzyl Alcohol	100-51-6	C7H8O	108.14	5.657	3.58	108.0566	79.0546	33.1	107.0498	62.0	Chlorobenzene-d5
Furaneol	3658-77-3	C6H8O3	128.13	5.738	17.42	128.0478	85.0294	38.9	---	---	Chlorobenzene-d5
Maltol	118-71-8	C6H6O3	126.11	6.132	8.74	126.0310	71.0136	15.2	---	---	Chlorobenzene-d5
L-Menthol	2216-51-5	C10H20O	156.26	6.334	6.31	123.1180	109.1024	37.7	138.1415	64.8	Chlorobenzene-d5
Methyl Salicylate	119-36-8	C8H8O3	152.15	6.489	4.52	120.0206	152.0469	79.1	---	---	Naphthalene-d8
Ethyl Maltol	4940-11-8	C7H8O3	140.14	6.597	6.75	140.0471	139.0399	32.1	---	---	Naphthalene-d8
(+)Pulegone	89-82-7	C10H16O	152.23	6.752	5.51	81.0706	152.1203	87.2	---	---	Naphthalene-d8
Ethyl Salicylate	118-61-6	C9H10O3	166.17	6.887	2.02	120.0204	166.0624	64.4	---	---	Naphthalene-d8
*trans*-Cinnamaldehyde	104-55-2	C9H8O	132.16	7.072	6.56	131.0499	132.0576	56.5	---	---	Naphthalene-d8
Triacetin	102-76-1	C9H14O6	218.20	7.230	8.86	103.0398	145.0506	62.8	---	---	Naphthalene-d8
Eugenol	97-53-0	C10H12O2	164.20	7.459	6.25	164.0830	149.0607	19.4	---	---	Naphthalene-d8
Vanillin	121-33-5	C8H8O3	152.15	8.130	14.91	152.0474	151.0399	55.4	---	---	Naphthalene-d8
Ethyl Vanillin	121-32-4	C9H10O3	166.17	8.571	0.33	137.0244	138.0321	66.6	---	---	Naphthalene-d8
Isovanillin	621-59-0	C8H8O3	152.50	8.747	11.91	152.0475	151.0400	59.4	---	---	Acenaphthene-d10
Internal Standards (IS)											
Benzene-d6	1076-43-3	C6H6	84.15	2.722	27.66	84.0851	85.0886	6.2	---	---	N/A
Pyridine-d5	7291-22-7	C5H5N	84.13	3.477	7.53	84.0739	56.0568	13.0	---	---	N/A
Chlorobenzene-d5	3114-55-4	C6H5Cl	117.59	4.184	42.81	117.0396	119.0369	29.5	---	---	N/A
Naphthalene-d8	1146-65-2	C10H8	136.22	6.478	0.58	136.1131	137.1168	10.1	---	---	N/A
Acenaphthene-d10	15067-26-2	C12H10	164.27	8.585	5.40	164.1416	165.1449	11.8	---	---	N/A

a Calculated using peak width (W) (determined from full width at half maximum height (FWHM)) and retention time (RT) from the peak immediately following, using the equation R_s_ = (RT_2_-RT_1_)/0.5(W_1_ +W_2_).

### Calibration

Coefficient of determination (*r* ^2^) values measured among the seven batches consistently exceeded 0.990 for all chemicals when fit using a weighted quadratic (1/x^2^) model (Supplementary Table S3, Supplemental Figure S1). Average RRF ranged from 0.03 (acetoin) to 0.70 (2,3,5-trimethylpyrazine) and RSE averaged below 10% for each chemical. Instrument linearity was established over the entire final concentration range (after 100-fold dilution) of 0.02 and 10 mg/ml for 20% of the chemicals. Benzyl alcohol, furaneol and *trans*-cinnamaldehyde reached detector saturation at a final concentration of 5 mg/ml, while the remaining chemicals met linearity up to 10 mg/ml but had varying lower limits (Supplementary Table S3). The lowest values in the instrument linear range were determined from average percent recovery and bias (CV) across seven calibrations (Supplementary Table S4). Quality control levels within the established instrumental range recovered within ±20% for each chemical. For 60% of the chemicals, the working calibration range met the same range as the instrument linear range. Butanoic acid, ethyl maltol, ethyl vanillin, isovanillin, maltol, *trans*-cinnamaldehyde, triacetin and vanillin each had tighter working calibration ranges (Supplementary Table S3), where additional lower levels were excluded based on results from the LLOQ and carryover method validation analyses.

### Method Validation

Fortified matrix samples (0.04, 0.10, 0.88, 1.75, 3.5 and 7.0 mg/ml) showed high precision and low bias (within ±20%) when compared within and between batches for 95% (19/20) of chemicals (Supplementary Table S5). Eucalyptol was within ±30% among three batches, while intra-day precision and bias remained within 20%. Greater variability was observed with recoveries of LLOQ fortified matrix samples (0.01, 0.04, 0.07, 0.10 and 0.27 mg/ml) where nine chemicals exceeded ±20% inter- and/or intra-batch precision (Supplementary Table S5). However, for seven of these chemicals, variability (±30%) was observed only in samples with concentrations below the instrumental linear range, where recoveries were considered estimated. Triacetin exceeded 30% inter- and intra-batch precision in 5/7 batches, resulting in a reduced working calibration range for this chemical. Bias across batches was within ±20% for all chemicals except eugenol, where two LLOQ fortified matrix samples (0.10 and 0.27 mg/ml) recovered within ±30% of the true concentration. Carryover was observed for butanoic acid, ethyl maltol, ethyl vanillin, isovanillin, maltol and vanillin when PG:VG blanks were analyzed proceeding concentrated samples (Supplementary Table S6). Likewise, ethyl maltol, isovanillin, maltol, *trans*-cinnamaldehyde and vanillin demonstrated carryover in methanol blanks. Accordingly, LLOQ values in the working calibration range for these chemicals were raised so that carryover accounted for <5% of the measured concentration. Dilutions of two- and 5-fold of each concentrated standard yielded intra-batch precision within ±20% for each chemical (Supplementary Table S7), except for benzyl alcohol and furaneol. Likewise, 10-fold dilutions of the 10 and 18.6 mg/ml samples were highly precise, with benzyl alcohol the sole chemical with higher variability between batches. Among the 50-fold dilutions, high precision was observed for 16/20 chemicals. Bias among all dilutions was within ±20% for 7/20 chemicals, while 14/20 were within ±30%. Stability over 1 month was observed in 18/20 chemicals where recovery was within 80–120%. Acetoin and furaneol presumably degraded, recovering below 80% of the expected concentration.

To assess the effect of the quadratic model on recovery, one acquisition batch was re-calculated after universally applying the linear (1/x^2^) model. Subsequent recovery of each calibration standard was then compared to the previously reported result. Sixteen chemicals had *r* ^2^ values exceeding 0.990 when fit using calibration standards within the working calibration range. Further, percent recoveries were within ±20% of the expected concentration for each calibration and QC standard. Compared to the average recoveries reported in Supplementary Table S4, CV was within ±20% for each standard of these 16 chemicals, with exception of the lowest calibration standard (0.04 mg/ml) for triacetin (25% CV). DL-limonene, eucalyptol, furaneol and *trans*-cinnamaldehyde had calculated *r* ^2^ values of 0.853, 0.985, 0.981 and 0.971 respectively, when calculated using a linear model. Concentrations of multiple standards when calculated against the linear fit were more variable, exceeding ±30% recovery and CV compared to quadratic recoveries.

To determine the variation between calibration curves analyzed in the same acquisition batch, percent drift was calculated using the opening calibration as the reference. Here, concentration of each calibration standard in the second calibration was subtracted from the corresponding standard from the first calibration and divided by the concentration of the first standard. The resulting drift for each chemical across concentrations within their respective working calibration ranges were within ±20% for all chemicals except dl-limonene (41% drift with 0.08 mg/ml standard), eucalyptol (69, 29 and 27% drift for 0.04, 0.16 and 0.31 mg/ml standards, respectively), benzyl alcohol (22% drift for 0.02 mg/ml standard), l-menthol (22% drift for 0.08 mg/ml standard) and pulegone (28% drift for 0.02 mg/ml standard).

### Flavoring Chemicals in Commercial E-Cigarette Liquids

**FIGURE 2:**
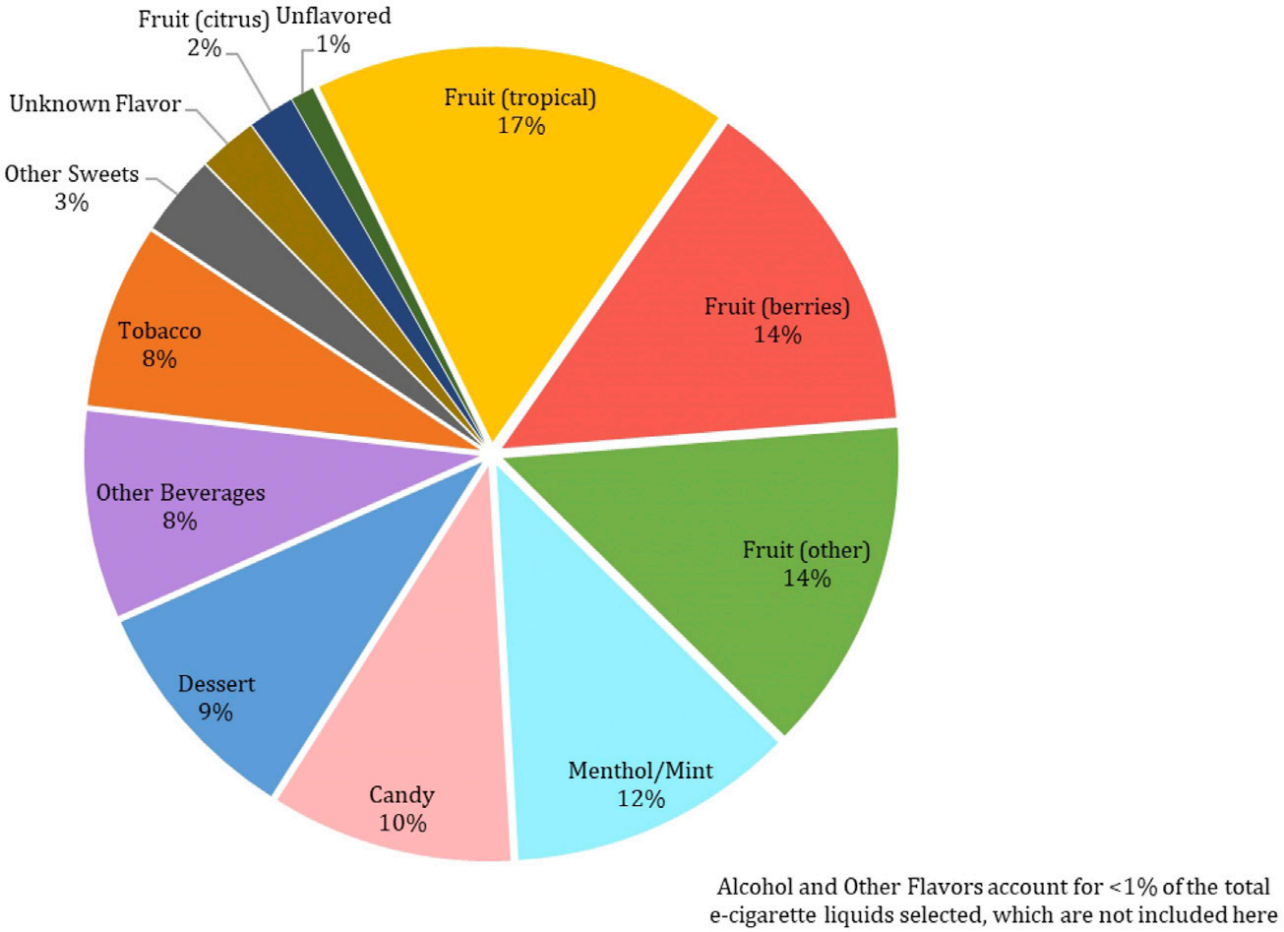
Frequency of 215 Selected Commercial Flavored E-Cigarette Liquids by Flavor Category.

**FIGURE 3:**
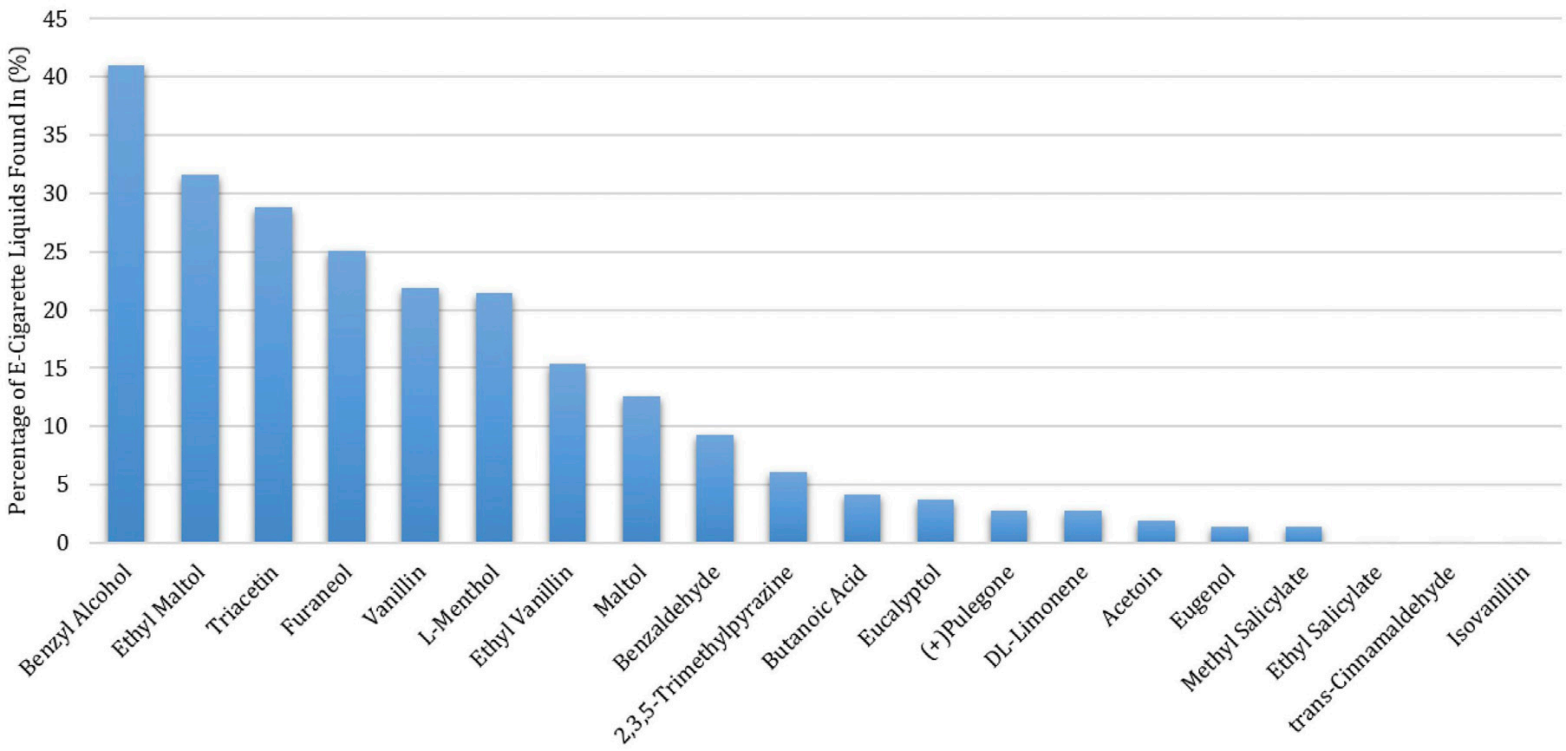
Percentage of Detections of Twenty Flavoring Chemicals Found in 215 Commercial Flavored E-Cigarette Liquids.

**FIGURE 4:**
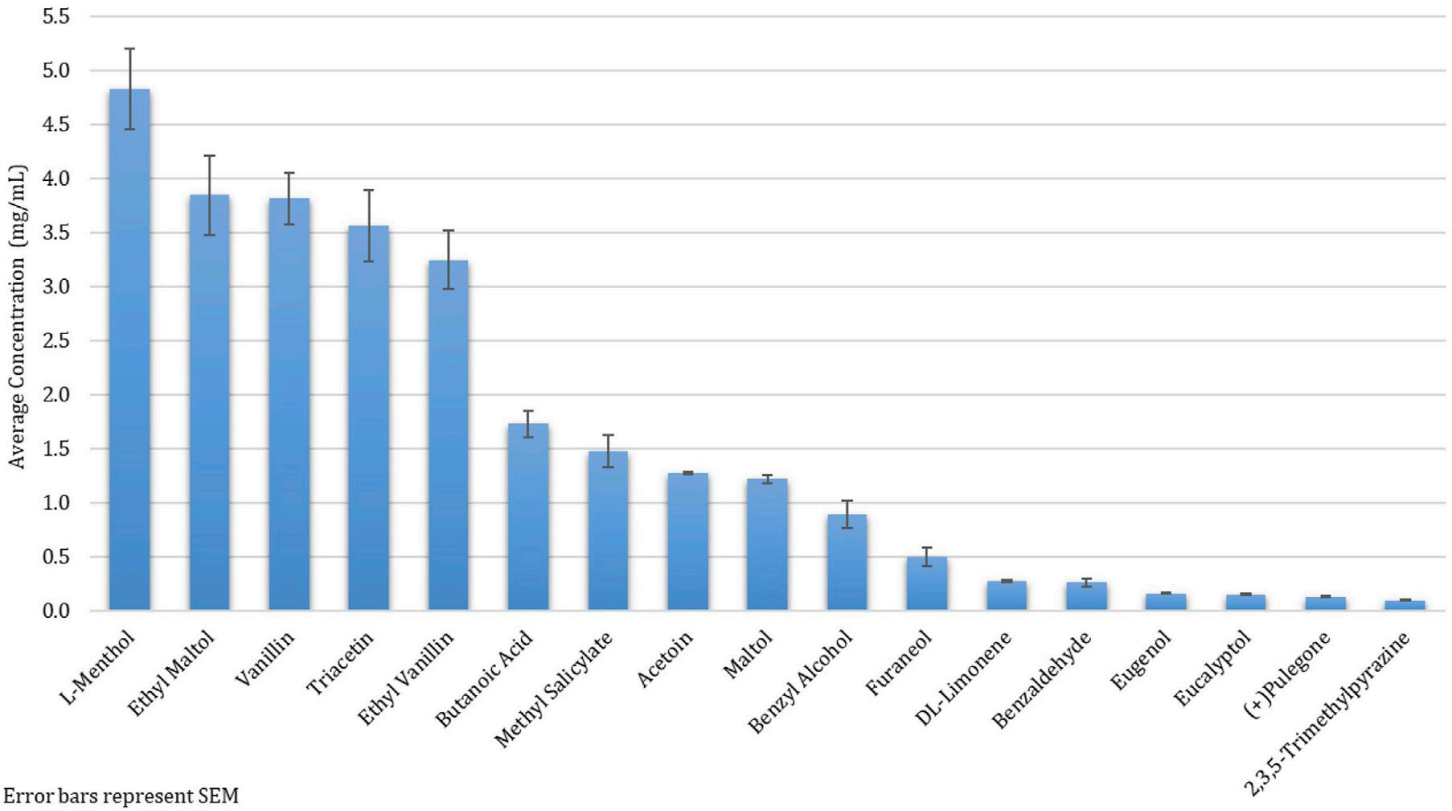
Average Concentration of Twenty Flavoring Chemicals found in 215 Selected Commercial Flavored E-Cigarette Liquids.

Among the 215 e-cigarette liquids selected, fruit flavors were most predominately analyzed, with tropical, berry and other-flavors (such as watermelon) comprising of 17, 14 and 13%, respectively of the total liquids (Figure 2). Between the remaining liquids, similar distributions across flavor categories were observed, where Menthol/Mint, Candy, Dessert, Other Beverages and Tobacco flavors encompassed 12, 10, 9, 8 and 8% of the liquids, respectively. All chemicals except for ethyl salicylate, isovanillin and *trans*-cinnamaldehyde were detected above the LLOQ in at least one e-cigarette liquid. Benzyl alcohol was the most abundant chemical found (in 41% of the products), followed by ethyl maltol (32%) and triacetin (29%) (Figure 3). L-menthol (detected in 21% of products) had the highest average concentration (4.83 mg/ml), followed by ethyl maltol (3.84 mg/ml), vanillin (3.81 mg/ml), triacetin (3.56 mg/ml) and ethyl vanillin (3.24 mg/ml) (Figure 4). Eight chemicals contained average concentrations below 1 mg/ml where 2,3,5-trimethylpyrazine averaged the lowest among liquids with 0.10 mg/ml. The highest individual concentrations were found with ethyl maltol (32.49 mg/ml, Candy-flavored), triacetin (23.15 mg/ml, Citrus fruit-flavored), ethyl vanillin (19.07 mg/ml, Other Sweets-flavored) and l-menthol (19.01 mg/ml, Menthol/Mint-flavored) (Supplementary Table S8). The lowest concentrations identified were 2,3,5-trimethylpyrazine and pulegone, where each were detected at 0.02 mg/ml. Several chemicals were identified in at least half of the liquids assigned to a single flavor category and included eugenol (100% in Fruit (tropical)), eucalyptol (100% in Menthol/Mint), pulegone (83% in Menthol/Mint), 2,3,5-trimethylpyrazine (62% in Tobacco), acetoin (50% in Dessert), and l-menthol (48% in Menthol/Mint).

## Discussion

The validated method demonstrates repeatable and accurate measurement of 20 commonly added flavoring chemicals of potential inhalation concern, where precision and accuracy of flavoring chemicals across batches were consistently within 20%. The large dynamic concentration range provides sensitivity for multiple chemicals in a single injection, while improving efficiency by reducing the frequency of re-analyses owing to overrange concentrations. As identified with this study, concentrations can vary by at least 1000-fold. Nearly 500 detections were calculated and less than 5% of the measured e-cigarette liquids required subsequent dilution. Further, our results suggest benzyl alcohol, ethyl maltol, ethyl vanillin, l-menthol, triacetin and vanillin are more likely to be added to e-cigarette liquids in concentrations greater than 10 mg/ml. This method has demonstrated precision and accuracy within ±20% across multiple dilutions for these six chemicals, with exception of benzyl alcohol. Here, values greater than 20% are found predominately with dilutions of the 10 mg/ml sample, suggesting a preparation issue with this chemical.

Quadratic calibration models were selected for several chemicals, rather than using a curve splitting technique for wide calibration concentration ranges (Basu et al., 2012) resulting in expediting data review. This model was universally applied throughout the method validation process to all chemicals for consistency. Calibration curves for many chemicals, however; were observed to follow linear trajectories (Supplemental Figure S1). To understand the difference in curve models, re-calculation of data in a single acquisition batch was performed using linear calibration curves for each chemical. After comparison to previous quadratic-fit data, concentrations of most chemicals (16/20) did not vary substantially, indicating minimal bias with the use of the quadratic model. Future assessment of the method should include the use of linear models for these chemicals. Recoveries of dl-limonene, eucalyptol, furaneol and *trans*-cinnamaldehyde varied more substantially after applying a linear model (without the use of curve splitting techniques), indicating a clear difference in instrumental response and suggestive of potential bias in the reported quadratic-based concentrations. However, recoveries from the fortified matrix samples at concentrations spanning the working calibration range were within ±20% of expected concentrations. Based on this validation, minimal bias is assumed with the quadratic model.

Benzyl alcohol, which had a reduced upper limit of quantitation, was relatively linear across validation batches. Two out of the seven acquisition batches used in the method validation experienced detector saturation beyond a final concentration of 5 mg/ml, for unknown reasons. Recoveries of benzyl alcohol from these batches were not excluded, leading to higher variability when assessing precision and bias. Based on these results, the upper level of quantitation (ULOQ) was lowered to 5 mg/ml. Subsequent analyses using this method has continued to show linearity beyond 5 mg/ml and method validation should be repeated for this chemical to establish high precision and low bias at a final concentration of 10 mg/ml.

Minimal carryover in methanol-only and PG/VG-only blanks injected immediately following the highest calibration standard was observed for six chemicals. Butanoic acid and ethyl vanillin appeared in PG/VG blanks only, suggesting that methanol alone, if used to assess carryover, is not sufficient to remove these chemicals from the system. Since e-cigarette liquids contain PG/VG, high concentrations of these chemicals may cause biased results in the subsequent injection. This has not been reported in previously published studies. To reduce this effect, LLOQ values were elevated such that reported concentrations were greater than 5% of possible carryover. Increased LLOQ values reduces the sensitivity of the method; however, several of these chemicals (ethyl maltol, ethyl vanillin and vanillin) were frequently found in higher concentrations, or not detected (isovanillin and *trans*-cinnamaldehyde) and are therefore not likely affected.

Multiple internal standards were included so that elution would span the chromatographic run and allow better representation of similarly eluting target chemicals. Relative retention times (RRT) of flavoring chemicals compared to their assigned internal standard were within the EPA suggested range of 0.80–1.20 (EPA, 2014) for 50% of the chemicals, while the furthest chemical was 1.51 (l-menthol) relative to its internal standard. Relative response factors (RRF) were calculated to assess the suitability of these assignments and selected concentration. Internal standard response should fall below 100 times the response of the target chemical, corresponding to a minimum value of 0.01 and an ideal value of 1 (EPA, 2014). Our method exceeds the minimum value for all chemicals, the lowest reaching 0.03 (acetoin). Validation batches exhibited consistent RRF values (data not shown), which further demonstrates the repeatability and stability of the instrument response over time.

The analytical column used in this method (DB-624 UI) was selected for the stationary phase, which is considered of intermediate polarity (Sigma-Aldrich, 2013) and designed for the rapid separation of volatile chemicals (Agilent_Products, 2021). Ultra Inert (UI) provides improved bonding and crosslinking of the stationary phase, leading to less column bleed and a lower baseline signal. Dimensions were selected to improve the efficiency of separations, which included a column length of 30 m and internal diameter of 0.25 mm (Rahman et al., 2015). Given the high concentrations of flavoring chemicals expected in e-cigarette liquids, a relatively large film thickness (1.4 μm) was selected to allow maximal loading capacity. Increased film thickness tends to increase peak width as chemical species are retained longer on the column, leading to reduced resolution, however; the chromatography observed in our method did not suffer.

This method takes advantage of innovative low-EI technology available in GC coupled MS instrumentation. Having a reduced applied ionization voltage (15 eV versus the traditional 70 eV) lowers the energy delivered to subsequent collisions of electrons with incoming chemical species and reduces the efficiency of ionization (Lau et al., 2019). This provides a two-fold advantage. First, given the high expected abundance of most flavoring chemicals in e-cigarette liquids, fewer chemical species are ionized, reducing potential detector saturation. Secondly, given the relatively small mass of the targeted flavoring chemicals, lower voltages may lead to softer fragmentation and increased abundance of the molecular ion. A comparison between ionization energies to understand fragmentation patterns of the flavoring chemicals was not performed in this study.

Detector sensitivity to matrix interference was not a concern in this method. The 7250 GC/Q-TOF, the newest of Q-TOF instrumentation from Agilent (currently), improves on the detector sensitivity of previous generations and has been utilized in low level detections of environmental contaminants. Here, the instrument has been shown to detect concentrations less than 10 ppb, while demonstrating sufficient separation from background noise (Agilent_Promotions, 2021). In contrast, the lowest detectable concentration required by our method is 2000-fold higher (20,000 ppb).

Few quantitative methods are available to measure concentrations of popular flavor additives in e-cigarette liquids, mainly using GC-MS (Bansal et al., 2019). Several studies followed a developed method containing 90 chemicals (Tierney et al., 2016) which was later expanded to 178 (Behar et al., 2018; Omaiye et al., 2019a; Omaiye et al., 2019b; Hua et al., 2019). Authentication standards were used to establish identifications; however, each study references the same published method for calibration procedures (Brown et al., 2014), where neither calibration or method validation information was provided. Further, this method was developed to measure chemicals in tobacco products and did not evaluate the e-cigarette liquid matrix. Likewise, an early published method used authentication standards to verify detections; however, only a three-point calibration was prepared (Schober et al., 2014) where concentrations were not reported. The wide range of concentrations found in their e-cigarette liquids combined with the lack of validation results yields uncertainty in the accuracy of the method. Conversely, the method published by Aszyk et al. includes a comprehensive method validation, offering bias and precision information for 46 chemicals, but excludes key flavoring chemicals with inhalation concern, such as ethyl maltol, ethyl vanillin, l-menthol, and vanillin (Aszyk et al., 2017). Important method parameters such as a reduced calibration range and use of acetonitrile limits time and cost effectiveness, which our method improves upon. Krüsemann et al. published a study which evaluated the validity of their method, but similarly had a limited calibration range (10-fold) and reduced target list (10 flavoring chemicals) (Krüsemann et al., 2020). In each of these published methods, the calibration curves have been prepared in the same organic solvent used to dilute the liquid. Our method is the first to prepare calibration levels using a similar matrix as the e-cigarette liquids themselves. Aszyk et al. evaluated matrix effects that impact the reported concentrations as part of their method validation. Our method accounts for this, therefore providing more accurate values for several chemicals identified with high matrix effects, such as benzyl alcohol (34%) and eugenol (133%) (Aszyk et al., 2017).

Our results confirm previous findings that high concentrations of several concerning flavoring chemicals are found in e-cigarette liquids (ethyl maltol, ethyl vanillin, triacetin, and vanillin). Further, pulegone was found almost exclusively in menthol/mint-flavored liquids at concentrations ranging from 0.02 to 0.32 mg/ml. Cinnamaldehyde, demonstrated to be highly cytotoxic (Behar et al., 2016) and disruptive to the immune response (Clapp et al., 2017), was not detected in any liquid tested in our study. This is not surprising since e-cigarette liquids with this characterizing cinnamon flavor were not included here. Our lower quantitation limits, however; allows for surveillance of such chemicals that may be added without the purpose of characterizing taste. For example, eugenol, with a flavor descriptor of spicy clove (The_Good_Scents_Company, 1980) is a common flavoring chemical found in clove cigarettes (Stanfill et al., 2006). While clove-flavored e-cigarette liquids were not included, this chemical was measured in low concentrations (<1 mg/ml) among several liquids exclusively characterized as tropical fruit-flavored. This is concerning as eugenol in clove cigarettes has been associated with pulmonary edema (LaVoie et al., 1986; Mcdonald and Heffner, 1991) and further acts as an anesthetic (Guidotti et al., 1989), allowing for deeper inhalation and more severe lung effects (infection and respiratory damage) (Hendee, 1988). Presence of this chemical in e-cigarette liquids, particularly in highly popular fruit flavors (Nguyen et al., 2019), may create a similar anesthetic effect, to which we have not identified published research relevant to e-cigarette users.

### Limitations

The stability of stock standards was not verified with newly purchased standards. Although storage followed vendor recommendations between use, the shelf-life of opened standards is generally unknown. This is particularly true of furaneol, where reactions with oxygen are visually observed with physical changes over time, despite storage under inert gas. Stability tests of calibration standards further demonstrates this loss, with less than 80% recovery after 1 month. Known degradation of acetoin was also observed after 1 month in calibration standards; however, the conversion to diacetyl was not assessed. Additionally, conversion of several aldehydes to their acetal forms was not determined with this method; however, acetals were observed qualitatively throughout the method validation process. Given the stability of the aldehydes in calibration solutions over 1 month, the conversion to acetals may be relatively quick as previously demonstrated (Erythropel et al., 2019). Reduced initial concentrations could lead to high biased measurements in e-cigarette liquids. Similarly, the e-cigarette liquids included here were previously obtained and degradation of flavoring chemicals are possible. Stability assessments of these 20 chemicals in commercial e-cigarette liquids is an ongoing project. Finally, the impact of PG/VG ratio in each calibration level is unknown. Since the initial level was prepared with nearly 50:50 methanol:PG/VG and increasing PG/VG volume for subsequent levels, the density differs between calibrators. However, linearity was established for all chemicals and multiple validation samples prepared with differing methanol to PG/VG ratios did not indicate substantial differences in recovery.

## Conclusion

Our newly developed method allows for the precise and accurate measurement of a wide range of concentrations for twenty flavoring chemicals of inhalation concern in commercial flavored e-cigarette liquids. With greater accuracy in the measurement among the liquid, the percentage of conversion to the aerosol and subsequent inhalation by the user can further assessed. This method can be applied to an assessment of inhalation exposure to flavoring chemicals in e-cigarette users.

## Data Availability

The raw data supporting the conclusions of this article will be made available by the authors, without undue reservation.
