# Supplementary material for: New Analytical Method for Quantifying Flavoring Chemicals of Potential Respiratory Health Risk Concerns in e-Cigarette Liquids
Source: Front Chem. 2021 Oct 28;9:763940. doi: 10.3389/fchem.2021.763940 (PMC8581464; doi:10.3389/fchem.2021.763940)
Supplement: Supplementary file 1 [file DataSheet1.pdf]

Supplemental Table 1 – Neat Standard Procurement and Storage Conditions

| Vendor | Standard | CAS | Purity (%) | Physical State | Storage Condition |
|---|---|---|---|---|---|
| Acros Organics | Butanoic Acid | 107-92-6 | 99+ | Liquid | Chemical Shelf |
| Acros Organics | Isovanillin | 621-59-0 | 98 | Solid | Chemical Shelf |
| Acros Organics | L-Limonene | 5989-54-8 | 96 | Liquid | Chemical Shelf |
| Acros Organics | L-Menthol | 2216-51-5 | 99.5 | Solid | Chemical Shelf |
| Acros Organics | Maltol | 118-71-8 | 99 | Solid | Chemical Shelf |
| Acros Organics | Triacetin | 102-76-1 | 99 | Liquid | Chemical Shelf |
| Alfa Aesar | Benzyl Alcohol | 100-51-6 | 99+ | Liquid | Chemical Shelf |
| Alfa Aesar | D-Limonene | 5989-27-5 | 97 | Liquid | Chemical Shelf |
| Alfa Aesar | Ethyl Vanillin | 121-32-4 | 98 | Solid | Chemical Shelf |
| Alfa Aesar | Eucalyptol | 470-82-6 | 99 | Liquid | Chemical Shelf |
| Alfa Aesar | Vanillin | 121-33-5 | 99 | Solid | Chemical Shelf |
| Cambridge Isotopes | Acenaphthene-d10 | 15067-26-2 | 99 | Solid | Chemical Shelf |
| Cambridge Isotopes | Benzene-d6 | 1076-43-3 | 99.5 | Liquid | Chemical Shelf |
| Cambridge Isotopes | Chlorobenzene-d5 | 3114-55-4 | 99 | Liquid | Chemical Shelf |
| Cambridge Isotopes | Pyridine-d5 | 7291-22-7 | 99.5 | Liquid | Chemical Shelf |
| Santa Cruz Biotechnology | Acetoin | 513-86-0 | 97 | Solid | 4-8°C |
| Santa Cruz Biotechnology | Naphthalene-d8 | 1146-65-2 | NA | Solid | Chemical Shelf |
| Sigma Aldrich | Benzaldehyde | 100-52-7 | 99.5 | Liquid | Chemical Shelf, under argon |
| Sigma Aldrich | Eugenol | 97-53-0 | 99 | Liquid | Chemical Shelf |
| Sigma Aldrich | Furaneol | 3658-77-3 | 99 | Solid | 4-8°C, under argon |
| Sigma Aldrich | Methyl Salicylate | 119-36-8 | 99 | Liquid | Chemical Shelf |
| TCI | (+)Pulegone | 89-82-7 | 95 | Liquid | Chemical Shelf |
| TCI | 2,3,5-Trimethylpyrazine | 14667-55-1 | 98 | Liquid | Chemical Shelf |
| TCI | Ethyl Maltol | 4940-11-8 | 99 | Solid | Chemical Shelf |
| TCI | Ethyl Salicylate | 118-61-6 | 99 | Liquid | Chemical Shelf |
| TCI | trans-Cinnamaldehyde | 14371-10-9 | 98 | Liquid | Chemical Shelf |

Supplemental Table 2 – Preparation Information for Calibration and Quality Control (QC) Standards

| Standard ID | Volume of Neat Standard (μL) | Volume of 20mg/mL Working Solution[2] (μL) | Volume of Previous Calibration Level (μL) | Volume of 50:50 PG:VG (5% H2O) (μL) | Total Volume (μL) | Concentration (mg/mL) |
|---|---|---|---|---|---|---|
| Initial Calibration | | | | | | |
| C10 | 100, each[1] | 5000 | NA | 3700 | 10,000 | 10.0 |
| C9 | --- | --- | 5000 (of C10) | 5000 | 10,000 | 5.00 |
| C8 | --- | --- | 5000 (of C9) | 5000 | 10,000 | 2.50 |
| C7 | --- | --- | 5000 (of C8) | 5000 | 10,000 | 1.25 |
| C6 | --- | --- | 5000 (of C7) | 5000 | 10,000 | 0.63 |
| C5 | --- | --- | 5000 (of C6) | 5000 | 10,000 | 0.31 |
| C4 | --- | --- | 5000 (of C5) | 5000 | 10,000 | 0.16 |
| C3 | --- | --- | 5000 (of C4) | 5000 | 10,000 | 0.08 |
| C2 | --- | --- | 5000 (of C3) | 5000 | 10,000 | 0.04 |
| C1 | --- | --- | 5000 (of C2) | 5000 | 10,000 | 0.02 |
| C0 | --- | --- | --- | 5000 | 10,000 | |
| Quality Control (QC) | | | | | | |
| QC9 | 80, each[1] | 4000 | NA | 4960 | 10,000 | 8.00 |
| QC8 | --- | --- | 5000 (of QC9) | 5000 | 10,000 | 4.00 |
| QC7 | --- | --- | 5000 (of QC8) | 5000 | 10,000 | 2.00 |
| QC6 | --- | --- | 5000 (of QC7) | 5000 | 10,000 | 1.00 |
| QC5 | --- | --- | 5000 (of QC6) | 5000 | 10,000 | 0.50 |
| QC4 | --- | --- | 5000 (of QC5) | 5000 | 10,000 | 0.25 |
| QC3 | --- | --- | 5000 (of QC4) | 5000 | 10,000 | 0.13 |
| QC2 | --- | --- | 5000 (of QC3) | 5000 | 10,000 | 0.06 |
| QC1 | --- | --- | 5000 (of QC2) | 5000 | 10,000 | 0.03 |

[1]Refer to Supplemental Table 1 for each liquid neat standard to be added

[2]Prepared by weighting 2.0000 ± 0.0005mg of each neat solid standard (see Supplemental Table 1) into 100mL of methanol and hand-vortex 5 min, or until visible granules are dissolved.

Supplemental Table 3 – Initial Calibration Parameters of Twenty Flavoring Chemicals

| | Instrument Linear Range[1] (mg/mL) | Working Calibration Range[2] (mg/mL) | Calibration Curve Fit | Average[3] Coefficient of Determination ($r^2$) | Average[3] RRF | Average[3] RRT | Average[3] RSE (%) | LLOQ (mg/mL) | S:N[4] at LLOQ |
|---|---|---|---|---|---|---|---|---|---|
| Acetoin | 0.31-10.00 | 0.31-10.00 | Quadratic, $1/x^2$ | 0.995 | 0.03 | 1.24 | 6.9 | 0.31 | 715 |
| Butanoic Acid | 0.16-10.00 | 0.63-10.00 | Quadratic, $1/x^2$ | 0.995 | 0.34 | 1.13 | 7.2 | 0.63 | 59 |
| Benzaldehyde | 0.02-10.00 | 0.02-10.00 | Quadratic, $1/x^2$ | 0.996 | 0.56 | 1.12 | 6.7 | 0.02 | 45 |
| 2,3,5-Trimethylpyrazine | 0.02-10.00 | 0.02-10.00 | Quadratic, $1/x^2$ | 0.995 | 0.70 | 1.24 | 7.9 | 0.02 | 846 |
| DL-Limonene | 0.08-10.00 | 0.08-10.00 | Quadratic, $1/x^2$ | 0.993 | 0.14 | 1.26 | 8.3 | 0.08 | 891 |
| Eucalyptol | 0.04-10.00 | 0.04-10.00 | Quadratic, $1/x^2$ | 0.995 | 0.09 | 1.29 | 7.0 | 0.04 | 438 |
| Benzyl Alcohol | 0.02-5.00 | 0.02-5.00 | Quadratic, $1/x^2$ | 0.995 | 0.54 | 1.35 | 7.1 | 0.02 | 164 |
| Furaneol | 0.02-5.00 | 0.02-5.00 | Quadratic, $1/x^2$ | 0.994 | 0.25 | 1.37 | 7.8 | 0.02 | 35 |
| Maltol | 0.16-10.00 | 0.63-10.00 | Quadratic, $1/x^2$ | 0.992 | 0.49 | 1.47 | 8.9 | 0.63 | 580 |
| L-Menthol | 0.08-10.00 | 0.08-10.00 | Quadratic, $1/x^2$ | 0.997 | 0.08 | 1.51 | 5.6 | 0.08 | 631 |
| Methyl Salicylate | 0.08-10.00 | 0.08-10.00 | Quadratic, $1/x^2$ | 0.993 | 0.36 | 1.00 | 8.5 | 0.08 | 2212 |
| Ethyl Maltol | 0.08-10.00 | 0.63-10.00 | Quadratic, $1/x^2$ | 0.992 | 0.24 | 1.02 | 8.4 | 0.63 | 492 |
| (+)Pulegone | 0.02-10.00 | 0.02-10.00 | Quadratic, $1/x^2$ | 0.995 | 0.19 | 1.04 | 6.5 | 0.02 | 1256 |
| Ethyl Salicylate | 0.02-10.00 | 0.02-10.00 | Quadratic, $1/x^2$ | 0.993 | 0.37 | 1.06 | 8.2 | 0.02 | 1986 |
| trans-Cinnamaldehyde | 0.02-5.00 | 0.16-5.00 | Quadratic, $1/x^2$ | 0.995 | 0.33 | 1.09 | 7.7 | 0.16 | 157 |
| Triacetin | 0.02-10.00 | 0.04-10.00 | Quadratic, $1/x^2$ | 0.997 | 0.18 | 1.12 | 5.7 | 0.04 | 10 |
| Eugenol | 0.04-10.00 | 0.04-10.00 | Quadratic, $1/x^2$ | 0.994 | 0.38 | 1.15 | 7.6 | 0.04 | 652 |
| Vanillin | 0.31-10.00 | 0.63-10.00 | Quadratic, $1/x^2$ | 0.994 | 0.24 | 1.26 | 7.9 | 0.63 | 159 |
| Ethyl Vanillin | 0.04-10.00 | 0.31-10.00 | Quadratic, $1/x^2$ | 0.995 | 0.14 | 1.32 | 7.3 | 0.31 | 1125 |
| Isovanillin | 0.04-10.00 | 0.31-10.00 | Quadratic, $1/x^2$ | 0.994 | 0.28 | 1.02 | 7.7 | 0.31 | 162 |

[1]Determined from percent recovery values (±20%) of each calibrator
[2]Determined from method validation parameters, including LLOQ and carryover analysis and represents calibration range applicable to quantitation of e-cigarette liquids
[3]Average of 7 independent batches analyzed across 7 days
[4]S:N = Signal/Noise ratio. Value calculated and provided by automated instrument software during data analysis. Acceptable peak identification from baseline noise is considered 10:1.

Supplemental Table 4 – Average Recovery and Coefficient of Variation (CV) Across 7 Calibration Batches

| | Initial Calibration | | | | | | | | | | Quality Control | | | | | | | | |
|---|---|---|---|---|---|---|---|---|---|---|---|---|---|---|---|---|---|---|---|
| Level | 1 | 2 | 3 | 4 | 5 | 6 | 7 | 8 | 9 | 10 | 1 | 2 | 3 | 4 | 5 | 6 | 7 | 8 | 9 |
| Expected Concentration (mg/mL) | 0.02 | 0.04 | 0.08 | 0.16 | 0.31 | 0.63 | 1.25 | 2.5 | 5.0 | 10.0 | 0.03 | 0.06 | 0.13 | 0.25 | 0.5 | 1.0 | 2.0 | 4.0 | 8.0 |
| | | | | | | | | | | | **Percent Recovery (%)[1]** | | | | | | | | |
| Acetoin | ***0*** | ***15*** | ***41*** | ***74*** | 101 | 97 | 99 | 102 | 100 | 100 | ***0*** | ***12*** | ***43*** | ***67*** | 102 | 100 | 100 | 101 | 97 |
| Butanoic Acid | ***30*** | ***26*** | *88* | 102 | 95 | 97 | 99 | 106 | 107 | 96 | ***25*** | ***26*** | ***78*** | 90 | 91 | 96 | 101 | 107 | 100 |
| Benzaldehyde | 99 | 99 | 95 | 98 | 98 | 100 | 106 | 107 | 104 | 94 | 91 | 94 | 93 | 93 | 95 | 100 | 102 | 102 | 96 |
| 2,3,5-Trimethylpyrazine | 105 | 96 | 94 | 95 | 96 | 99 | 109 | 110 | 104 | 91 | 95 | 90 | 90 | 91 | 94 | 101 | 105 | 102 | 92 |
| DL-Limonene | ***55*** | *90* | 97 | 99 | 99 | 98 | 99 | 98 | 108 | 97 | ***58*** | *81* | 102 | 100 | 105 | 103 | 102 | 111 | 105 |
| Eucalyptol | *87* | 99 | 99 | 100 | 98 | 100 | 101 | 101 | 102 | 99 | *90* | 99 | 96 | 98 | 99 | 98 | 98 | 102 | 96 |
| Benzyl Alcohol | 93 | 102 | 100 | 95 | 98 | 97 | 105 | 108 | 104 | ***67*** | 102 | 94 | 92 | 94 | 96 | 102 | 108 | 107 | ***71*** |
| Furaneol | 102 | 88 | 100 | 97 | 102 | 102 | 106 | 104 | 96 | ***13*** | 104 | 105 | 95 | 101 | 103 | 106 | 104 | 105 | ***14*** |
| Maltol | ***45*** | ***55*** | *86* | 93 | 94 | 96 | 104 | 111 | 108 | 93 | ***43*** | ***56*** | ***79*** | 90 | 94 | 100 | 107 | 109 | 98 |
| L-Menthol | ***73*** | *82* | 100 | 100 | 101 | 98 | 102 | 101 | 101 | 99 | ***61*** | *87* | 99 | 99 | 98 | 100 | 99 | 101 | 100 |
| Methyl Salicylate | *85* | *85* | 95 | 93 | 97 | 100 | 107 | 111 | 106 | 92 | *82* | *81* | 94 | 95 | 97 | 104 | 107 | 107 | 96 |
| Ethyl Maltol | ***45*** | ***71*** | 100 | 91 | 95 | 96 | 104 | 109 | 110 | 94 | ***42*** | ***65*** | 95 | 92 | 94 | 103 | 109 | 113 | 100 |
| (+)Pulegone | 98 | 99 | 103 | 95 | 100 | 97 | 101 | 104 | 106 | 96 | 104 | 94 | 103 | 98 | 98 | 101 | 103 | 104 | 99 |
| Ethyl Salicylate | 99 | 95 | 94 | 97 | 97 | 100 | 107 | 112 | 107 | 92 | 98 | 93 | 95 | 93 | 98 | 105 | 109 | 110 | 97 |
| trans-Cinnamaldehyde | 99 | 98 | 97 | 97 | 101 | 101 | 107 | 104 | 94 | ***14*** | 103 | 97 | 95 | 97 | 100 | 104 | 106 | 102 | ***14*** |
| Triacetin | 100 | 98 | 98 | 99 | 98 | 100 | 101 | 104 | 104 | 97 | 93 | 94 | 95 | 93 | 96 | 96 | 99 | 102 | 97 |
| Eugenol | *86* | 95 | 95 | 95 | 97 | 100 | 106 | 110 | 108 | 92 | *87* | 96 | 94 | 96 | 99 | 106 | 110 | 112 | 99 |
| Vanillin | ***71*** | ***70*** | ***70*** | *81* | 96 | 101 | 101 | 105 | 108 | 95 | ***67*** | ***65*** | ***66*** | *80* | 94 | 98 | 104 | 109 | 100 |
| Ethyl Vanillin | *91* | 95 | 97 | 96 | 96 | 100 | 103 | 106 | 108 | 95 | *86* | 99 | 95 | 94 | 95 | 102 | 104 | 110 | 99 |
| Isovanillin | *90* | 98 | 97 | 95 | 96 | 100 | 104 | 106 | 107 | 94 | *92* | 95 | 92 | 95 | 99 | 101 | 104 | 108 | 97 |
| Benzene-d6 | 107 | 104 | 103 | 103 | 102 | 103 | 100 | 101 | 101 | 101 | 95 | 95 | 94 | 95 | 94 | 95 | 95 | 94 | 96 |
| Pyridine-d5 | 105 | 104 | 102 | 102 | 102 | 101 | 100 | 100 | 100 | 100 | 97 | 98 | 97 | 96 | 96 | 96 | 94 | 95 | 95 |
| Chlorobenzene-d5 | 107 | 105 | 101 | 102 | 103 | 103 | 100 | 101 | 101 | 102 | 97 | 98 | 97 | 98 | 97 | 97 | 97 | 97 | 97 |
| Naphthalene-d8 | 106 | 104 | 101 | 101 | 101 | 102 | 100 | 100 | 98 | 97 | 96 | 97 | 96 | 96 | 97 | 96 | 95 | 94 | 93 |
| Acenaphthene-d10 | 107 | 105 | 102 | 102 | 103 | 102 | 100 | 101 | 100 | 99 | 98 | 98 | 98 | 98 | 97 | 98 | 98 | 96 | 95 |
| | | | | | | | | | | | **Coefficient of Variation (CV) (%)[1]** | | | | | | | | |
| Acetoin | ***N/A*** | ***265*** | ***125*** | ***69*** | 6 | 7 | 6 | 4 | 3 | 2 | ***N/A*** | ***265*** | ***126*** | ***70*** | 14 | 6 | 5 | 4 | 4 |

| | | | | | | | | | | | | | | | | | | | |
|---|---|---|---|---|---|---|---|---|---|---|---|---|---|---|---|---|---|---|---|
| Butanoic Acid | ***171*** | ***171*** | ***46*** | 6 | 4 | 5 | 3 | 2 | 3 | 2 | ***171*** | ***171*** | ***45*** | 9 | 5 | 5 | 3 | 3 | 2 |
| Benzaldehyde | 6 | 8 | 5 | 3 | 3 | 4 | 3 | 2 | 2 | 3 | 10 | 7 | 7 | 4 | 4 | 3 | 4 | 2 | 4 |
| 2,3,5-Trimethylpyrazine | 3 | 6 | 2 | 4 | 3 | 2 | 1 | 2 | 2 | 4 | 7 | 7 | 3 | 5 | 3 | 3 | 3 | 4 | 4 |
| DL-Limonene | ***95*** | ***46*** | 9 | 10 | 6 | 3 | 2 | 4 | 3 | 2 | ***95*** | ***46*** | 7 | 7 | 8 | 8 | 7 | 5 | 2 |
| Eucalyptol | ***44*** | 13 | 8 | 9 | 7 | 4 | 3 | 4 | 3 | 2 | ***46*** | 8 | 8 | 6 | 4 | 3 | 2 | 4 | 2 |
| Benzyl Alcohol | 8 | 6 | 4 | 6 | 3 | 3 | 1 | 3 | 5 | ***68*** | 11 | 11 | 6 | 5 | 6 | 5 | 4 | 4 | ***68*** |
| Furaneol | 5 | 7 | 8 | 3 | 4 | 2 | 4 | 5 | 7 | ***265*** | 8 | 8 | 9 | 6 | 3 | 2 | 2 | 3 | ***265*** |
| Maltol | ***125*** | ***94*** | ***44*** | 6 | 3 | 4 | 3 | 3 | 2 | 2 | ***125*** | ***94*** | ***44*** | 4 | 6 | 5 | 3 | 3 | 3 |
| L-Menthol | ***68*** | ***45*** | 7 | 6 | 6 | 6 | 4 | 3 | 3 | 2 | ***70*** | ***47*** | 9 | 5 | 3 | 5 | 2 | 3 | 4 |
| Methyl Salicylate | ***46*** | ***44*** | 6 | 3 | 3 | 3 | 4 | 2 | 2 | 3 | ***44*** | ***45*** | 6 | 4 | 2 | 2 | 4 | 2 | 4 |
| Ethyl Maltol | ***125*** | ***69*** | 5 | 5 | 4 | 5 | 2 | 2 | 2 | 1 | ***125*** | ***68*** | 6 | 5 | 4 | 5 | 1 | 3 | 1 |
| (+)Pulegone | 7 | 9 | 7 | 5 | 3 | 3 | 3 | 3 | 2 | 2 | 12 | 7 | 7 | 4 | 4 | 2 | 2 | 4 | 1 |
| Ethyl Salicylate | 7 | 5 | 7 | 3 | 2 | 3 | 2 | 1 | 3 | 2 | 4 | 4 | 5 | 3 | 2 | 3 | 1 | 3 | 3 |
| trans-Cinnamaldehyde | 10 | 8 | 4 | 3 | 4 | 3 | 4 | 2 | 6 | ***265*** | 11 | 6 | 5 | 3 | 3 | 2 | 2 | 5 | ***265*** |
| Triacetin | 6 | 8 | 4 | 6 | 4 | 4 | 2 | 2 | 2 | 1 | 15 | 11 | 5 | 2 | 4 | 3 | 2 | 3 | 1 |
| Eugenol | ***44*** | 6 | 6 | 1 | 4 | 2 | 4 | 2 | 2 | 3 | ***45*** | 3 | 4 | 2 | 3 | 4 | 2 | 3 | 2 |
| Vanillin | ***70*** | ***68*** | ***69*** | ***44*** | 2 | 4 | 6 | 4 | 3 | 3 | ***69*** | ***69*** | ***68*** | ***44*** | 6 | 6 | 5 | 4 | 3 |
| Ethyl Vanillin | ***45*** | 10 | 5 | 8 | 3 | 4 | 3 | 2 | 3 | 1 | ***48*** | 6 | 6 | 4 | 2 | 5 | 2 | 3 | 4 |
| Isovanillin | ***45*** | 8 | 7 | 5 | 4 | 3 | 3 | 2 | 4 | 3 | ***45*** | 7 | 6 | 5 | 4 | 8 | 4 | 3 | 2 |
| Benzene-d6 | 4 | 6 | 4 | 3 | 3 | 4 | 0 | 2 | 2 | 3 | 4 | 6 | 2 | 4 | 3 | 4 | 4 | 3 | 3 |
| Pyridine-d5 | 5 | 6 | 4 | 5 | 3 | 3 | 0 | 3 | 2 | 2 | 3 | 4 | 4 | 3 | 4 | 4 | 4 | 3 | 3 |
| Chlorobenzene-d5 | 6 | 8 | 4 | 3 | 2 | 1 | 0 | 3 | 2 | 1 | 3 | 5 | 3 | 4 | 2 | 4 | 3 | 2 | 3 |
| Naphthalene-d8 | 4 | 5 | 2 | 2 | 3 | 3 | 0 | 2 | 2 | 2 | 2 | 3 | 2 | 2 | 2 | 4 | 3 | 3 | 3 |
| Acenaphthene-d10 | 7 | 7 | 3 | 3 | 3 | 3 | 0 | 3 | 2 | 3 | 3 | 2 | 2 | 3 | 2 | 4 | 3 | 2 | 2 |

[1]Averaged from 7 independent batches analyzed across 7 days

Italicized and grey indicates values outside of the instrument linear range where recovery is estimated. Bold indicates values outside of 80-120% percent recovery or ±20% CV.

Supplemental Table 5 – Method Validation Parameters for Mid-Range and Low-Range Fortified Matrix Samples Across 7 Batches

| Method Validation Measure[1] | Mid-Range Fortified Matrix Samples | | | | | | LLOQ Fortified Matrix Samples | | | | |
|---|---|---|---|---|---|---|---|---|---|---|---|
| | Expected Concentration (mg/mL) | | | | | | | | | | |
| | 0.04 | 0.10 | 0.88 | 1.75 | 3.5 | 7.0 | 0.01 | 0.04 | 0.07 | 0.10 | 0.27 |
| **Acetoin** | | | | | | | | | | | |
| ***Within Batch Precision (%)*** | | | | | | | | | | | |
| *Batch # 1* | 8 | 19 | 4 | 7 | 4 | 2 | --- | **21** | **29** | 7 | 5 |
| *2* | **27** | 4 | 9 | 6 | 3 | 1 | --- | **33** | 3 | 20 | 11 |
| *3* | 9 | 8 | 3 | 4 | 3 | 4 | --- | 8 | 4 | 9 | 10 |
| *4* | 6 | **38** | 4 | 6 | 1 | 3 | --- | **41** | **50** | 19 | 14 |
| *5* | --- | 5 | 6 | 2 | 6 | 7 | --- | --- | --- | 20 | 15 |
| *6* | --- | **92** | 5 | 6 | 2 | 5 | --- | 5 | --- | --- | 9 |
| *7* | --- | --- | 7 | 4 | 9 | 2 | --- | --- | 5 | **29** | 4 |
| ***Between Batch Precision (%)*** | **68** | **33** | 6 | 6 | 6 | 5 | --- | **50** | **53** | **29** | 12 |
| ***Between Batch Bias (%)*** | **37** | 17 | 2 | 1 | 1 | 1 | --- | **48** | 0 | 9 | 5 |
| **Butanoic Acid** | | | | | | | | | | | |
| ***Within Batch Precision (%)*** | | | | | | | | | | | |
| *Batch # 1* | 8 | 7 | 5 | 5 | 3 | 1 | 19 | 5 | 6 | 9 | 3 |
| *2* | 7 | 10 | 2 | 2 | 3 | 2 | 2 | 5 | 5 | 11 | 2 |
| *3* | 10 | 5 | 0 | 2 | 3 | 2 | 7 | 14 | 7 | 8 | 6 |
| *4* | 4 | 6 | 0 | 1 | 1 | 4 | 7 | 6 | 5 | 5 | 2 |
| *5* | 6 | 3 | 1 | 2 | 2 | 2 | 20 | 8 | 0 | 2 | 5 |
| *6* | 17 | 10 | 4 | 3 | 2 | 1 | **51** | 19 | 14 | 10 | 7 |
| *7* | 11 | 7 | 3 | 3 | 3 | 3 | --- | 29 | 9 | 3 | 7 |
| ***Between Batch Precision (%)*** | **32** | 14 | 4 | 3 | 3 | 5 | **43** | 30 | 17 | 14 | 8 |
| ***Between Batch Bias (%)*** | **39** | -2 | -8 | -2 | 4 | 1 | **252** | 36 | 4 | -4 | -13 |
| **Benzaldehyde** | | | | | | | | | | | |
| ***Within Batch Precision (%)*** | | | | | | | | | | | |
| *Batch # 1* | 11 | 2 | 5 | 2 | 4 | 5 | 18 | 7 | 4 | 4 | 0 |
| *2* | 6 | 4 | 1 | 3 | 2 | 4 | 15 | 3 | 4 | 1 | 5 |
| *3* | 2 | 3 | 1 | 2 | 3 | 4 | 10 | 4 | 6 | 4 | 4 |
| *4* | 4 | 7 | 3 | 5 | 1 | 1 | 18 | 2 | 3 | 5 | 6 |
| *5* | 11 | 6 | 1 | 1 | 2 | 3 | **21** | 6 | 7 | 13 | 2 |
| *6* | 13 | 4 | 2 | 1 | 3 | 1 | **28** | 1 | 8 | 4 | 5 |
| *7* | 8 | 2 | 1 | 2 | 4 | 0 | 4 | 9 | 4 | 3 | 5 |
| ***Between Batch Precision (%)*** | 8 | 6 | 4 | 5 | 4 | 4 | **30** | 12 | 8 | 7 | 6 |

| | | | | | | | | | | | | |
|---|---|---|---|---|---|---|---|---|---|---|---|---|
| ***Between Batch Bias (%)*** | | -5 | -10 | -5 | -1 | 0 | -2 | *-5* | -5 | -6 | -5 | -5 |
| **2,3,5-Trimethylpyrazine** | | | | | | | | | | | | |
| ***Within Batch Precision (%)*** | | | | | | | | | | | | |
| *Batch #* | *1* | 3 | 2 | 1 | 1 | 1 | 4 | *2* | 7 | 7 | 6 | 2 |
| | *2* | 4 | 1 | 2 | 2 | 1 | 2 | *5* | 0 | 5 | 1 | 2 |
| | *3* | 7 | 4 | 2 | 2 | 2 | 2 | *7* | 9 | 2 | 4 | 3 |
| | *4* | 4 | 3 | 2 | 4 | 0 | 2 | *9* | 11 | 3 | 8 | 0 |
| | *5* | 1 | 4 | 2 | 2 | 0 | 2 | *5* | 6 | 3 | 2 | 1 |
| | *6* | 2 | 2 | 0 | 1 | 1 | 2 | *13* | 6 | 5 | 7 | 2 |
| | *7* | 5 | 5 | 3 | 2 | 3 | 2 | *4* | 5 | 6 | 7 | 2 |
| ***Between Batch Precision (%)*** | | 6 | 5 | 2 | 3 | 3 | 8 | *18* | 9 | 7 | 6 | 3 |
| ***Between Batch Bias (%)*** | | 2 | -9 | 1 | 7 | 7 | 0 | *19* | -4 | -7 | -6 | -8 |
| **DL-Limonene** | | | | | | | | | | | | |
| ***Within Batch Precision (%)*** | | | | | | | | | | | | |
| *Batch #* | *1* | *6* | 1 | 3 | 7 | 2 | 3 | *3* | *3* | 9 | 5 | 5 |
| | *2* | *12* | 9 | 0 | 1 | 1 | 2 | *5* | *9* | 9 | 7 | 7 |
| | *3* | *18* | 10 | 3 | 1 | 3 | 1 | *17* | *9* | 7 | 11 | 8 |
| | *4* | *12* | 12 | 5 | 5 | 1 | 1 | ***89*** | *19* | 5 | 8 | 10 |
| | *5* | *3* | 11 | 6 | 2 | 2 | 1 | *19* | *3* | 13 | 9 | 1 |
| | *6* | *14* | 8 | 5 | 2 | 2 | 1 | *---* | ***33*** | 17 | 9 | 10 |
| | *7* | *12* | 6 | 3 | 3 | 0 | 1 | *8* | *9* | 14 | 5 | 6 |
| ***Between Batch Precision (%)*** | | *14* | 10 | 10 | 6 | 4 | 3 | ***46*** | *19* | 13 | 9 | 10 |
| ***Between Batch Bias (%)*** | | ***32*** | 11 | -2 | -17 | -5 | 12 | ***66*** | *11* | 1 | 1 | -6 |
| **Eucalyptol** | | | | | | | | | | | | |
| ***Within Batch Precision (%)*** | | | | | | | | | | | | |
| *Batch #* | *1* | **22** | 12 | 2 | 2 | 2 | 4 | ***28*** | **26** | 9 | 17 | 11 |
| | *2* | 10 | 7 | 4 | 1 | 1 | 2 | *19* | 12 | 11 | 2 | 7 |
| | *3* | 8 | 5 | 3 | 5 | 2 | 1 | *10* | **22** | 8 | 8 | 2 |
| | *4* | 13 | 4 | 1 | 1 | 1 | 2 | ***36*** | 1 | 3 | 12 | 10 |
| | *5* | **24** | 4 | 2 | 4 | 2 | 1 | ***48*** | 11 | 25 | 2 | 5 |
| | *6* | **25** | 15 | 3 | 3 | 2 | 0 | ***76*** | 18 | 16 | 14 | 3 |
| | *7* | 18 | 9 | 2 | 6 | 1 | 2 | ***146*** | 6 | 13 | 7 | 7 |
| ***Between Batch Precision (%)*** | | 19 | 9 | 3 | 5 | 4 | 4 | ***62*** | 19 | 13 | 10 | 8 |
| ***Between Batch Bias (%)*** | | 4 | -1 | -1 | -2 | 0 | -1 | ***-32*** | -13 | -7 | -6 | -8 |
| **Benzyl Alcohol** | | | | | | | | | | | | |
| ***Within Batch Precision (%)*** | | | | | | | | | | | | |
| *Batch #* | *1* | 8 | 6 | 4 | 1 | 4 | *3* | ***23*** | 1 | 5 | 8 | 5 |
| | *2* | 11 | 6 | 4 | 7 | 1 | *1* | *5* | 9 | 10 | 3 | 1 |
| | *3* | 14 | 6 | 7 | 2 | 1 | *2* | *14* | 4 | 5 | 2 | 7 |

| | | | | | | | | | | | | |
|---|---|---|---|---|---|---|---|---|---|---|---|---|
| | 4 | 4 | 4 | 1 | 2 | 1 | 2 | 11 | 11 | 2 | 6 | 3 |
| | 5 | 5 | 13 | 4 | 1 | 1 | 2 | 22 | 6 | 6 | 2 | 1 |
| | 6 | 4 | 4 | 5 | 3 | 1 | 2 | 13 | 9 | 6 | 2 | 3 |
| | 7 | 16 | 3 | 3 | 2 | 3 | 2 | 4 | 8 | 2 | 1 | 2 |
| ***Between Batch Precision (%)*** | | 13 | 11 | 7 | 5 | 4 | 9 | 21 | 13 | 14 | 9 | 11 |
| ***Between Batch Bias (%)*** | | -1 | -7 | 2 | 8 | 8 | -2 | 12 | 1 | 2 | 0 | 0 |
| **Furaneol** | | | | | | | | | | | | |
| ***Within Batch Precision (%)*** | | | | | | | | | | | | |
| *Batch #* | 1 | 11 | 8 | 4 | 3 | 4 | 5 | 22 | 15 | 10 | 10 | 5 |
| | 2 | 11 | 4 | 4 | 4 | 7 | --- | 2 | 7 | 3 | 3 | 5 |
| | 3 | 9 | 5 | 2 | 4 | 7 | 24 | 7 | 9 | 4 | 6 | 1 |
| | 4 | 14 | 1 | 3 | 4 | 6 | --- | 18 | 13 | 5 | 5 | 5 |
| | 5 | 10 | 9 | 6 | 6 | 10 | --- | 18 | 14 | 9 | 2 | 7 |
| | 6 | 19 | 5 | 5 | 2 | 4 | --- | 10 | 8 | 4 | 11 | 2 |
| | 7 | 4 | 3 | 4 | 4 | 13 | --- | 35 | 9 | 3 | 8 | 2 |
| ***Between Batch Precision (%)*** | | 13 | 5 | 4 | 4 | 7 | 17 | 21 | 12 | 9 | 9 | 5 |
| ***Between Batch Bias (%)*** | | 6 | -3 | 3 | 5 | 8 | 7 | 7 | -4 | -7 | -7 | -8 |
| **Maltol** | | | | | | | | | | | | |
| ***Within Batch Precision (%)*** | | | | | | | | | | | | |
| *Batch #* | 1 | 5 | 5 | 0 | 0 | 4 | 3 | 1 | 1 | 4 | 5 | 3 |
| | 2 | 3 | 5 | 5 | 3 | 4 | 2 | 8 | 2 | 3 | 4 | 3 |
| | 3 | 4 | 2 | 3 | 2 | 2 | 3 | 2 | 3 | 3 | 0 | 3 |
| | 4 | 9 | 4 | 1 | 2 | 1 | 0 | 5 | 5 | 5 | 3 | 3 |
| | 5 | 5 | 1 | 3 | 1 | 2 | 1 | 4 | 4 | 7 | 2 | 5 |
| | 6 | 7 | 4 | 2 | 2 | 2 | 2 | 12 | 8 | 6 | 3 | 2 |
| | 7 | 9 | 8 | 3 | 4 | 3 | 2 | 9 | 2 | 3 | 5 | 5 |
| ***Between Batch Precision (%)*** | | 37 | 16 | 4 | 3 | 3 | 5 | 67 | 38 | 23 | 16 | 5 |
| ***Between Batch Bias (%)*** | | 42 | -2 | -3 | 5 | 9 | 3 | 217 | 34 | 2 | -6 | -14 |
| **L-Menthol** | | | | | | | | | | | | |
| ***Within Batch Precision (%)*** | | | | | | | | | | | | |
| *Batch #* | 1 | 27 | 16 | 5 | 2 | 3 | 4 | 65 | 17 | 4 | 10 | 11 |
| | 2 | 13 | 9 | 1 | 1 | 4 | 3 | 39 | 16 | 7 | 6 | 3 |
| | 3 | 14 | 10 | 2 | 3 | 1 | 3 | 34 | 27 | 29 | 11 | 7 |
| | 4 | 6 | 13 | 6 | 4 | 1 | 1 | 53 | 33 | 7 | 13 | 9 |
| | 5 | 4 | 9 | 1 | 4 | 3 | 2 | 96 | 2 | 7 | 26 | 12 |
| | 6 | 10 | 8 | 2 | 2 | 2 | 1 | 88 | 4 | 16 | 8 | 7 |
| | 7 | 17 | 14 | 4 | 4 | 2 | 3 | --- | 20 | 25 | 13 | 3 |
| ***Between Batch Precision (%)*** | | 18 | 12 | 4 | 3 | 4 | 4 | 114 | 29 | 22 | 17 | 8 |
| ***Between Batch Bias (%)*** | | 8 | 0 | -2 | -1 | 0 | 1 | -29 | 6 | 0 | -5 | -8 |

| | | | | | | | | | | | | |
|---|---|---|---|---|---|---|---|---|---|---|---|---|
| | | | | | | **Methyl Salicylate** | | | | | | |
| ***Within Batch Precision (%)*** | | | | | | | | | | | | |
| *Batch #* | *1* | 6 | 6 | 2 | 3 | 1 | 2 | **22** | 8 | 8 | 7 | 3 |
| | *2* | 2 | 4 | 0 | 1 | 1 | 1 | 14 | 10 | 3 | 1 | 3 |
| | *3* | 10 | 2 | 1 | 2 | 1 | 1 | 9 | 5 | 3 | 5 | 3 |
| | *4* | 4 | 5 | 3 | 3 | 1 | 1 | 4 | 2 | 5 | 3 | 6 |
| | *5* | 2 | 4 | 2 | 3 | 4 | 1 | 7 | 7 | 5 | 3 | 1 |
| | *6* | 2 | 2 | 2 | 1 | 2 | 2 | 14 | 6 | 3 | 4 | 8 |
| | *7* | 15 | 8 | 3 | 2 | 1 | 2 | 3 | 12 | 3 | 7 | 6 |
| ***Between Batch Precision (%)*** | | 18 | 7 | 3 | 3 | 2 | 3 | **54** | **22** | 11 | 9 | 5 |
| ***Between Batch Bias (%)*** | | 6 | -4 | 0 | 4 | 7 | 0 | **35** | -3 | -7 | -9 | -11 |
| | | | | | | **Ethyl Maltol** | | | | | | |
| ***Within Batch Precision (%)*** | | | | | | | | | | | | |
| *Batch #* | *1* | 2 | 7 | 2 | 1 | 1 | 1 | 4 | 5 | 9 | 8 | 4 |
| | *2* | 1 | 10 | 3 | 2 | 4 | 0 | 9 | 3 | 3 | 9 | 3 |
| | *3* | 3 | 6 | 2 | 2 | 1 | 1 | 5 | 2 | 3 | 7 | 3 |
| | *4* | 7 | 6 | 1 | 4 | 2 | 1 | 4 | 2 | 11 | 6 | 2 |
| | *5* | 9 | 3 | 4 | 3 | 2 | 1 | 5 | 3 | 7 | 8 | 4 |
| | *6* | 1 | 6 | 2 | 3 | 2 | 1 | 13 | 5 | 5 | 5 | 4 |
| | *7* | 4 | 8 | 3 | 2 | 3 | 2 | 8 | 10 | 4 | 1 | 7 |
| ***Between Batch Precision (%)*** | | 18 | 8 | 5 | 5 | 4 | 3 | **40** | **22** | 14 | 10 | 5 |
| ***Between Batch Bias (%)*** | | **25** | -6 | -2 | 7 | 11 | 3 | **148** | 12 | -9 | -13 | -18 |
| | | | | | | **(+)Pulegone** | | | | | | |
| ***Within Batch Precision (%)*** | | | | | | | | | | | | |
| *Batch #* | *1* | 6 | 11 | 1 | 3 | 1 | 3 | **26** | 13 | 13 | 9 | 5 |
| | *2* | 4 | 3 | 3 | 2 | 1 | 0 | 9 | 6 | 4 | 4 | 2 |
| | *3* | 5 | 5 | 2 | 2 | 0 | 1 | 13 | 5 | 4 | 4 | 3 |
| | *4* | 15 | 4 | 4 | 3 | 1 | 2 | 4 | 6 | 6 | 3 | 3 |
| | *5* | 12 | 3 | 2 | 3 | 6 | 1 | 9 | 13 | 6 | 9 | 6 |
| | *6* | 1 | 7 | 2 | 3 | 2 | 1 | 13 | 5 | 5 | 9 | 5 |
| | *7* | 12 | 13 | 1 | 1 | 2 | 3 | 17 | 4 | 10 | 7 | 0 |
| ***Between Batch Precision (%)*** | | 9 | 7 | 3 | 3 | 3 | 3 | 17 | 9 | 9 | 7 | 5 |
| ***Between Batch Bias (%)*** | | 1 | -7 | -4 | -1 | 2 | 0 | 7 | -4 | -10 | -7 | -7 |
| | | | | | | **Ethyl Salicylate** | | | | | | |
| ***Within Batch Precision (%)*** | | | | | | | | | | | | |
| *Batch #* | *1* | 7 | 6 | 1 | 1 | 0 | 1 | 2 | 13 | 8 | 7 | 3 |
| | *2* | 10 | 4 | 0 | 2 | 1 | 1 | 5 | 1 | 6 | 4 | 3 |
| | *3* | 1 | 5 | 4 | 1 | 0 | 2 | 8 | 3 | 2 | 5 | 2 |
| | *4* | 4 | 4 | 1 | 1 | 2 | 1 | 7 | 8 | 8 | 1 | 4 |

| | | | | | | | | | | | |
|---|---|---|---|---|---|---|---|---|---|---|---|
| *5* | 7 | 4 | 2 | 0 | 3 | 1 | *13* | 5 | 2 | 6 | 4 |
| *6* | 10 | 3 | 1 | 3 | 3 | 2 | *6* | 3 | 10 | 4 | 3 |
| *7* | 13 | 4 | 2 | 2 | 1 | 1 | *10* | 4 | 4 | 4 | 4 |
| ***Between Batch Precision (%)*** | 7 | 5 | 2 | 2 | 2 | 3 | *18* | 8 | 6 | 5 | 4 |
| ***Between Batch Bias (%)*** | -2 | -6 | 0 | 4 | 8 | 1 | *8* | -9 | -10 | -11 | -10 |
| **trans-Cinnamaldehyde** | | | | | | | | | | | |
| ***Within Batch Precision (%)*** | | | | | | | | | | | |
| *Batch # 1* | 4 | 4 | 4 | 4 | 4 | *1* | *2* | 4 | 5 | 2 | 4 |
| *2* | 3 | 7 | 2 | 5 | 5 | --- | ***23*** | 10 | 2 | 7 | 4 |
| *3* | 1 | 4 | 4 | 2 | 5 | *13* | *18* | 13 | 4 | 4 | 3 |
| *4* | 9 | 5 | 2 | 1 | 8 | *13* | *8* | 11 | 11 | 3 | 4 |
| *5* | 5 | 5 | 2 | 2 | 11 | --- | *7* | 2 | 5 | 8 | 5 |
| *6* | 9 | 8 | 5 | 6 | 7 | --- | *6* | 11 | 3 | 5 | 3 |
| *7* | 9 | 4 | 2 | 2 | 11 | --- | ***29*** | 9 | 4 | 9 | 4 |
| ***Between Batch Precision (%)*** | 9 | 6 | 5 | 5 | 8 | *11* | *19* | 10 | 6 | 7 | 5 |
| ***Between Batch Bias (%)*** | -3 | -13 | -5 | -2 | -1 | *-18* | *9* | -6 | -7 | -9 | -8 |
| **Triacetin** | | | | | | | | | | | |
| ***Within Batch Precision (%)*** | | | | | | | | | | | |
| *Batch # 1* | 14 | 3 | 1 | 3 | 2 | 2 | ***28*** | 16 | 2 | 4 | 6 |
| *2* | 3 | 4 | 4 | 1 | 2 | 1 | ***40*** | 4 | 3 | 5 | 5 |
| *3* | 8 | 6 | 3 | 3 | 2 | 2 | ***33*** | 11 | 5 | 3 | 3 |
| *4* | 16 | 8 | 5 | 4 | 1 | 3 | ***36*** | 9 | 7 | 9 | 0 |
| *5* | 12 | 12 | 4 | 3 | 3 | 1 | *16* | **22** | 8 | 5 | 4 |
| *6* | 20 | 8 | 6 | 1 | 3 | 1 | ***40*** | 12 | 7 | 9 | 9 |
| *7* | 4 | 4 | 5 | 3 | 3 | 1 | ***45*** | 4 | 12 | 4 | 8 |
| ***Between Batch Precision (%)*** | 16 | 7 | 4 | 3 | 3 | 3 | ***41*** | 19 | 11 | 8 | 8 |
| ***Between Batch Bias (%)*** | -6 | -7 | -4 | -1 | 1 | -2 | *-7* | -8 | -11 | -8 | -13 |
| **Eugenol** | | | | | | | | | | | |
| ***Within Batch Precision (%)*** | | | | | | | | | | | |
| *Batch # 1* | 0 | 4 | 4 | 3 | 2 | 3 | *7* | 8 | 2 | 8 | 2 |
| *2* | 1 | 6 | 1 | 2 | 1 | 3 | *3* | 7 | 3 | 4 | 3 |
| *3* | 3 | 4 | 1 | 3 | 2 | 3 | *4* | 4 | 3 | 4 | 2 |
| *4* | 4 | 5 | 2 | 3 | 3 | 2 | *13* | 4 | 7 | 8 | 7 |
| *5* | 10 | 5 | 1 | 2 | 5 | 1 | *11* | 7 | 10 | 5 | 4 |
| *6* | 5 | 4 | 3 | 1 | 1 | 2 | *5* | 8 | 6 | 4 | 4 |
| *7* | 7 | 2 | 2 | 1 | 3 | 3 | *13* | 13 | 8 | 3 | 0 |
| ***Between Batch Precision (%)*** | 11 | 5 | 3 | 3 | 3 | 3 | ***30*** | 14 | 8 | 6 | 4 |
| ***Between Batch Bias (%)*** | -1 | -10 | -1 | 5 | 9 | 0 | *13* | -15 | -20 | **-21** | **-22** |
| **Vanillin** | | | | | | | | | | | |

| | | | | | | | | | | | |
|---|---|---|---|---|---|---|---|---|---|---|---|
| ***Within Batch Precision (%)*** | | | | | | | | | | | |
| *Batch #* *1* | *4* | *2* | 1 | 1 | 4 | 3 | *4* | *5* | *3* | *5* | 4 |
| *2* | *10* | *9* | 1 | 2 | 1 | 2 | *5* | *5* | *8* | *9* | 3 |
| *3* | *4* | *4* | 3 | 3 | 1 | 3 | ***41*** | *14* | *7* | *5* | 7 |
| *4* | *5* | *3* | 3 | 3 | 1 | 1 | *4* | *9* | *3* | *3* | 7 |
| *5* | *18* | *3* | 4 | 3 | 3 | 1 | ***21*** | *17* | *2* | *5* | 2 |
| *6* | *11* | *2* | 1 | 2 | 3 | 1 | *10* | *2* | *7* | *7* | 5 |
| *7* | *16* | *3* | 3 | 1 | 4 | 2 | *10* | *6* | *3* | *2* | 5 |
| ***Between Batch Precision (%)*** | ***35*** | *13* | 6 | 7 | 5 | 6 | ***86*** | ***41*** | ***21*** | *14* | 9 |
| ***Between Batch Bias (%)*** | ***31*** | *-3* | -5 | 1 | 7 | 2 | ***97*** | *17* | *-3* | *-11* | -20 |
| **Ethyl Vanillin** | | | | | | | | | | | |
| ***Within Batch Precision (%)*** | | | | | | | | | | | |
| *Batch #* *1* | 2 | 7 | 1 | 3 | 2 | 3 | ***21*** | 10 | 7 | 21 | 2 |
| *2* | 16 | 7 | 2 | 4 | 0 | 2 | *8* | 17 | 1 | 2 | 4 |
| *3* | 9 | 4 | 2 | 3 | 1 | 1 | ***35*** | 9 | 9 | 4 | 5 |
| *4* | 4 | 6 | 5 | 2 | 2 | 2 | *13* | 19 | 9 | 9 | 1 |
| *5* | 16 | 7 | 4 | 2 | 4 | 1 | ***75*** | 6 | 8 | 9 | 4 |
| *6* | 9 | 2 | 2 | 2 | 2 | 2 | *20* | 4 | 13 | 12 | 10 |
| *7* | 13 | 4 | 2 | 3 | 3 | 2 | *9* | 8 | 7 | 5 | 5 |
| ***Between Batch Precision (%)*** | 13 | 6 | 3 | 3 | 3 | 4 | ***47*** | 15 | 9 | 11 | 10 |
| ***Between Batch Bias (%)*** | 5 | -6 | 1 | 5 | 9 | 2 | *9* | -7 | -7 | -16 | -15 |
| **Isovanillin** | | | | | | | | | | | |
| ***Within Batch Precision (%)*** | | | | | | | | | | | |
| *Batch #* *1* | 14 | 2 | 3 | 3 | 2 | 4 | *5* | 8 | 3 | 4 | 8 |
| *2* | 1 | 1 | 1 | 5 | 6 | 4 | *10* | 4 | 10 | 5 | 3 |
| *3* | 2 | 7 | 4 | 5 | 7 | 2 | ***57*** | 4 | 8 | 6 | 4 |
| *4* | 11 | 8 | 5 | 6 | 5 | 6 | ***39*** | 11 | 3 | 6 | 2 |
| *5* | 9 | 8 | 1 | 5 | 2 | 6 | *14* | 5 | 11 | 3 | 3 |
| *6* | 3 | 1 | 4 | 2 | 5 | 6 | ***27*** | 8 | 3 | 11 | 2 |
| *7* | 11 | 3 | 6 | 1 | 6 | 3 | ***23*** | 16 | 7 | 5 | 1 |
| ***Between Batch Precision (%)*** | 12 | 5 | 5 | 4 | 5 | 5 | ***49*** | 10 | 9 | 7 | 6 |
| ***Between Batch Bias (%)*** | 1 | -7 | 1 | 5 | 8 | 1 | *11* | -6 | -14 | -12 | -12 |

[1]Averaged from 7 independent batches analyzed across 7 days

Italicized and grey indicates values outside of the instrument linear range where recovery is estimated. Bold indicates values outside of 80-120% percent recovery or ±20% CV.

Supplemental Table 6 – Carryover Assessment with 50:50 PG:VG and Methanol Matrix Blanks

| *Matrix blank assessed* | 50:50 PG:VG | | Methanol | |
|---|---|---|---|---|
| *Standard concentration injected prior to matrix blank* | 8mg/mL | 10mg/mL | 8mg/mL | 10mg/mL |
| ***Average Carryover (mg/mL)*** | | | | |
| Acetoin | 0.00 | 0.00 | 0.00 | 0.00 |
| Butanoic Acid | **0.04** | **0.04** | 0.00 | 0.00 |
| Benzaldehyde | 0.00 | 0.00 | 0.00 | 0.00 |
| 2,3,5-Trimethylpyrazine | 0.00 | 0.00 | 0.00 | 0.00 |
| DL-Limonene | 0.00 | 0.00 | 0.00 | 0.00 |
| Eucalyptol | 0.00 | 0.00 | 0.00 | 0.00 |
| Benzyl Alcohol | 0.00 | 0.00 | 0.00 | 0.00 |
| Furaneol | 0.00 | 0.00 | 0.00 | 0.00 |
| Maltol | **0.04** | **0.04** | **0.03** | **0.03** |
| L-Menthol | 0.00 | 0.00 | 0.00 | 0.00 |
| Methyl Salicylate | 0.00 | 0.00 | 0.00 | 0.00 |
| Ethyl Maltol | **0.03** | **0.03** | **0.02** | **0.01** |
| (+)Pulegone | 0.00 | 0.00 | 0.00 | 0.00 |
| Ethyl Salicylate | 0.00 | 0.00 | 0.00 | 0.00 |
| trans-Cinnamaldehyde | 0.00 | 0.00 | **0.01** | **0.01** |
| Triacetin | 0.00 | 0.00 | 0.00 | 0.00 |
| Eugenol | 0.00 | 0.00 | 0.00 | 0.00 |
| Vanillin | **0.05** | 0.00 | **0.04** | 0.00 |
| Ethyl Vanillin | **0.01** | 0.00 | 0.00 | 0.00 |
| Isovanillin | 0.00 | **0.01** | **0.02** | **0.01** |

Supplemental Table 7 – Dilution Precision and Bias Determined from Four Dilution Factors

| *Dilution Factor* | 2X | | | 5X | | | 10X | | 50X | |
|---|---|---|---|---|---|---|---|---|---|---|
| *Fortified Matrix Sample Concentration (mg/mL)* | 5 | 10 | 18.6 | 5 | 10 | 18.6 | 10 | 18.6 | 10 | 18.6 |
| *Concentration after dilution (mg/mL)* | 2.5 | 5 | 9.3 | 1 | 2 | 3.72 | 1 | 1.86 | 0.2 | 0.372 |
| ***Between Run Precision (CV%)*** | | | | | | | | | | |
| Acetoin | 7 | 7 | 2 | 7 | 9 | 6 | 11 | 6 | *17* | 17 |
| Butanoic Acid | 14 | 16 | 19 | 15 | 13 | 4 | 20 | 5 | 16 | 14 |
| Benzaldehyde | 3 | 2 | 6 | 3 | 5 | 6 | 9 | 5 | 8 | 11 |
| 2,3,5-Trimethylpyrazine | 5 | 6 | 10 | 7 | 6 | 5 | 7 | 4 | 9 | 16 |
| DL-Limonene | 10 | 7 | 16 | 13 | 13 | 14 | 19 | 8 | 11 | 7 |
| Eucalyptol | 4 | 3 | 10 | 4 | 5 | 4 | 9 | 7 | 7 | 8 |
| Benzyl Alcohol | 10 | 4 | *14* | **26** | 18 | 7 | **23** | **21** | **41** | **46** |
| Furaneol | 11 | 17 | ***28*** | 11 | 19 | **27** | 18 | 17 | 10 | **23** |
| Maltol | 4 | 6 | 5 | 3 | 6 | 3 | 6 | 6 | 9 | 10 |
| L-Menthol | 3 | 5 | 10 | 4 | 5 | 4 | 6 | 6 | 5 | 14 |
| Methyl Salicylate | 4 | 3 | 9 | 6 | 4 | 4 | 7 | 6 | 6 | 16 |
| Ethyl Maltol | 4 | 4 | 3 | 5 | 6 | 5 | 7 | 7 | 12 | 13 |
| (+)Pulegone | 5 | 2 | 9 | 6 | 6 | 4 | 5 | 5 | 8 | 17 |
| Ethyl Salicylate | 3 | 5 | 10 | 3 | 3 | 5 | 6 | 5 | 6 | 13 |
| trans-Cinnamaldehyde | 4 | 12 | --- | 9 | 15 | 13 | 19 | 17 | 15 | **23** |
| Triacetin | 4 | 4 | 8 | 7 | 6 | 5 | 7 | 6 | 13 | 20 |
| Eugenol | 4 | 4 | 7 | 5 | 5 | 4 | 6 | 3 | 8 | 17 |
| Vanillin | 9 | 7 | 9 | 7 | 7 | 8 | 8 | 9 | *16* | 19 |
| Ethyl Vanillin | 5 | 6 | 3 | 8 | 4 | 6 | 5 | 7 | 14 | 17 |
| Isovanillin | 5 | 2 | 19 | 5 | 4 | 13 | 9 | 15 | 11 | **23** |
| ***Between Run Bias (CV%)*** | | | | | | | | | | |
| Acetoin | 9 | 6 | -2 | 7 | 1 | -7 | 17 | -10 | 19 | -14 |
| Butanoic Acid | -11 | -5 | -14 | -17 | -14 | -3 | -14 | -4 | -14 | **-33** |
| Benzaldehyde | 16 | 16 | 0 | 14 | 16 | 7 | **25** | 6 | 16 | -10 |
| 2,3,5-Trimethylpyrazine | 14 | 8 | -5 | 10 | 10 | 3 | **21** | 5 | 13 | -15 |
| DL-Limonene | **26** | **28** | -9 | 11 | **42** | 8 | **41** | 1 | **45** | 4 |
| Eucalyptol | 5 | 7 | -9 | 3 | 5 | -9 | 14 | -10 | 8 | **-23** |
| Benzyl Alcohol | **24** | 10 | *-12* | **33** | **25** | 6 | **47** | 20 | **54** | 19 |
| Furaneol | -11 | **-21** | ***-37*** | -18 | **-26** | **-42** | -14 | **-38** | -11 | **-39** |

| | | | | | | | | | | |
|---|---|---|---|---|---|---|---|---|---|---|
| Maltol | 11 | 13 | -3 | 0 | 9 | 5 | 14 | 1 | -2 | **-26** |
| L-Menthol | 5 | 9 | -4 | 5 | 7 | -4 | **22** | 1 | **29** | -6 |
| Methyl Salicylate | 13 | 12 | -10 | 8 | 17 | 3 | **24** | 5 | 15 | -13 |
| Ethyl Maltol | 13 | 13 | -6 | 2 | 11 | 0 | 13 | -4 | -3 | **-28** |
| (+)Pulegone | 9 | 7 | -9 | 6 | 6 | -5 | 17 | -2 | 18 | -11 |
| Ethyl Salicylate | 20 | 14 | -9 | 16 | **21** | 6 | **31** | 10 | 20 | -9 |
| trans-Cinnamaldehyde | 15 | 9 | -8 | 7 | -4 | -13 | 7 | -14 | 19 | -17 |
| Triacetin | 6 | 5 | -8 | 4 | 6 | -7 | 15 | -5 | 16 | -15 |
| Eugenol | 15 | 10 | -8 | 9 | 12 | 1 | 19 | 3 | 12 | -14 |
| Vanillin | -1 | -8 | 7 | -5 | -13 | 1 | -7 | 2 | *-8* | -15 |
| Ethyl Vanillin | 6 | -5 | 11 | 1 | -7 | 8 | 2 | 9 | -1 | -6 |
| Isovanillin | -2 | -18 | **53** | -4 | **-26** | 20 | **-21** | 17 | -19 | 1 |

Italicized and grey indicates values outside of the instrument linear range where recovery is estimated. Bold indicates values outside of ±20% CV.

Supplemental Table 8 – Descriptive Summary of Twenty Flavoring Chemicals Detected in 215 Commercial E-Cigarette Liquids with Various Flavors

| | n | Average Concentration (mg/mL) | Highest concentration (mg/mL) | Lowest concentration (mg/mL) | Median Concentration (mg/mL) | Flavor Category Predominately Found | % Found in most Predominate Flavor Category |
|---|---|---|---|---|---|---|---|
| L-Menthol | 46 | 4.83 | 19.01 | 0.09 | 2.30 | Menthol/Mint | 48 |
| Ethyl Maltol | 68 | 3.84 | 32.49 | 0.51 | 2.10 | Fruit (tropical), Dessert | 24, 19 |
| Vanillin | 47 | 3.81 | 16.22 | 0.63 | 2.67 | Dessert | 35 |
| Triacetin | 62 | 3.56 | 23.15 | 0.08 | 1.86 | Fruit (tropical), Fruit (berries) | 27, 16 |
| Ethyl Vanillin | 33 | 3.24 | 19.07 | 0.35 | 1.65 | Dessert | 36 |
| Butanoic Acid | 9 | 1.73 | 6.34 | 0.71 | 1.03 | Fruit (berries) | 44 |
| Methyl Salicylate | 3 | 1.47 | 4.01 | 0.18 | 0.23 | Menthol/Mint, Fruit (tropical), Tobacco | 33 each |
| Acetoin | 4 | 1.27 | 1.52 | 1.12 | 1.22 | Dessert | 50 |
| Maltol | 27 | 1.22 | 2.63 | 0.64 | 1.03 | Fruit (berries), Fruit (other) | 30, 26 |
| Benzyl Alcohol | 88 | 0.89 | 16.05 | 0.02 | 0.23 | Fruit (tropical), Fruit (berries) | 26, 22 |
| Furaneol | 54 | 0.50 | 8.47 | 0.02 | 0.19 | Candy, Dessert, Fruit (berries) | 22, 20, 19 |
| Benzaldehyde | 20 | 0.26 | 2.51 | 0.02 | 0.06 | Candy, Other Beverages | 35, 25 |
| DL-Limonene | 7 | 0.24 | 0.48 | 0.04 | 0.25 | Menthol/Mint | 33 |
| Eugenol | 3 | 0.16 | 0.31 | 0.07 | 0.10 | Fruit (tropical) | 100 |
| Eucalyptol | 8 | 0.15 | 0.42 | 0.04 | 0.12 | Menthol/Mint | 100 |
| (+)Pulegone | 6 | 0.13 | 0.32 | 0.02 | 0.11 | Menthol/Mint | 83 |
| 2,3,5-Trimethylpyrazine | 13 | 0.10 | 0.31 | 0.02 | 0.05 | Tobacco | 62 |
| Ethyl Salicylate | --- | --- | --- | --- | --- | --- | --- |
| trans-Cinnamaldehyde | --- | --- | --- | --- | --- | --- | --- |
| Isovanillin | --- | --- | --- | --- | --- | --- | --- |

Supplemental Figure 1 – Calibration Curves for Twenty Flavoring Chemicals

(A) Acetoin

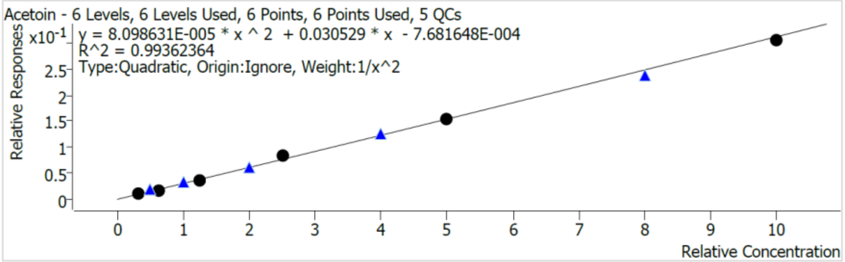


(B) Butanoic Acid

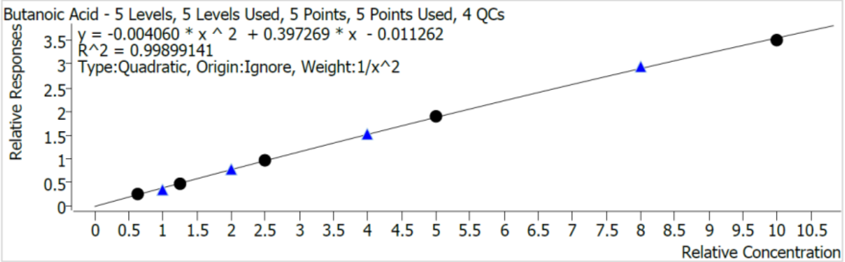


(C) Benzaldehyde

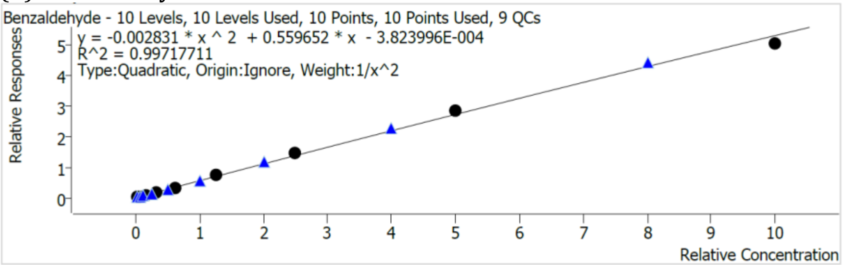


(D) 2,3,5-Trimethylpyrazine

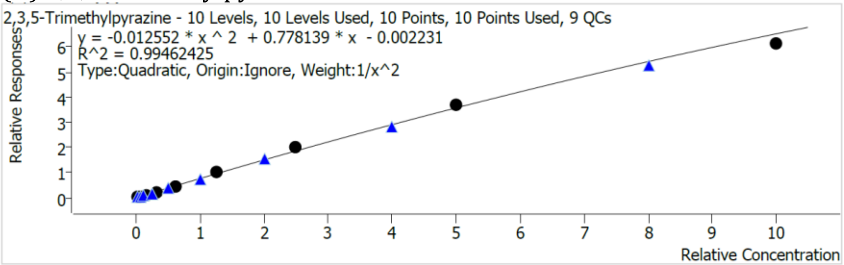


(E) DL-Limonene

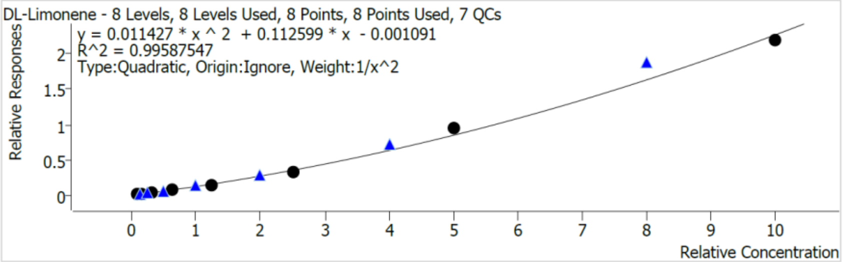


(F) Eucalyptol

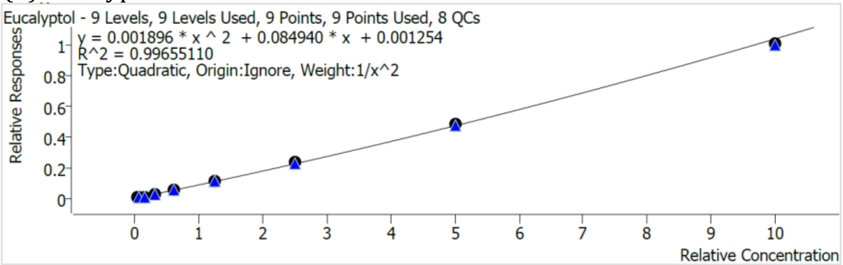


(G) Benzyl Alcohol

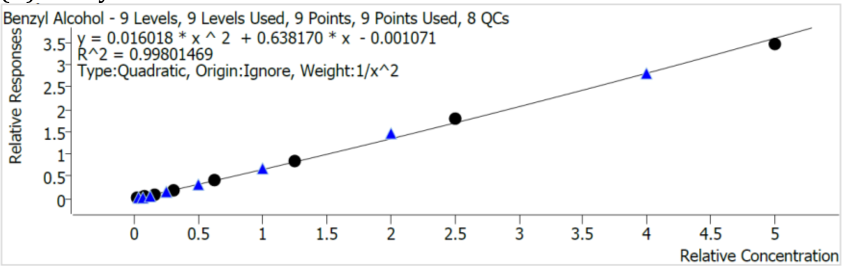


(H) Furaneol

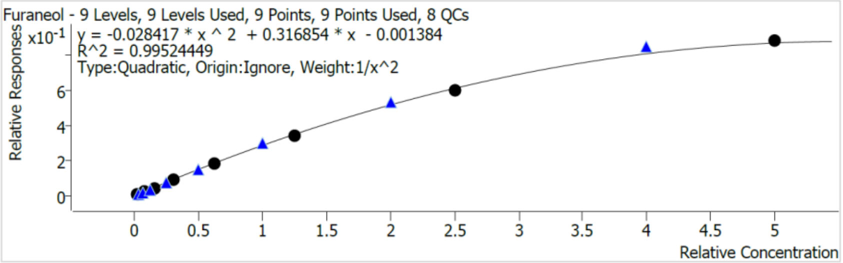

(I) Maltol

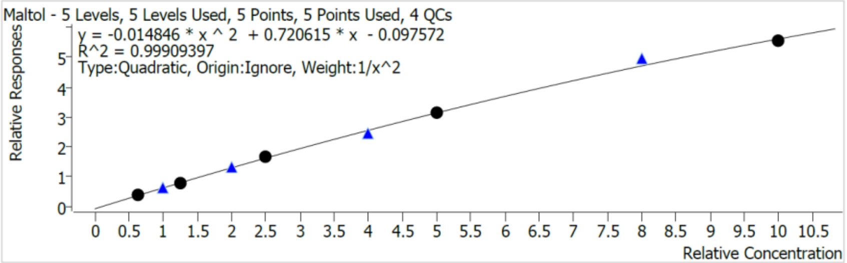


(J) L-Menthol

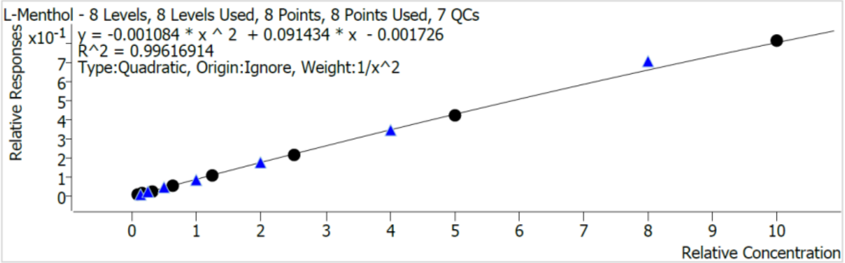


(K) Methyl Salicylate

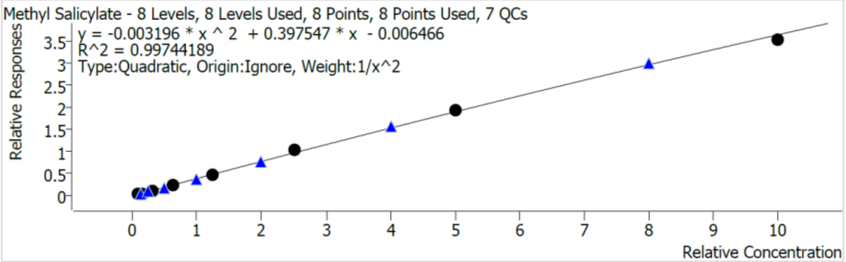


(L) Ethyl Maltol

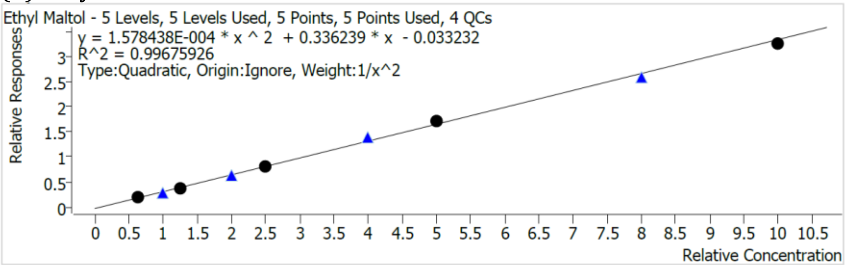


(M) (+)Pulegone

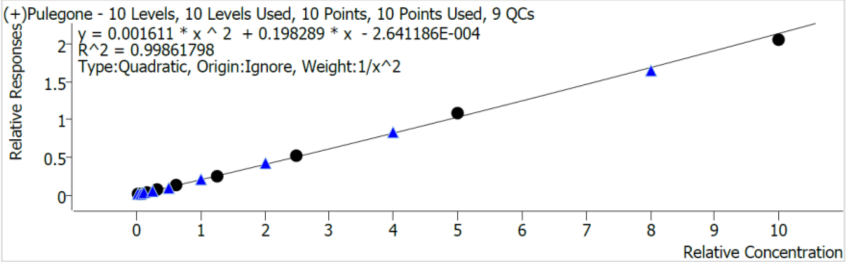


(N) Ethyl Salicylate

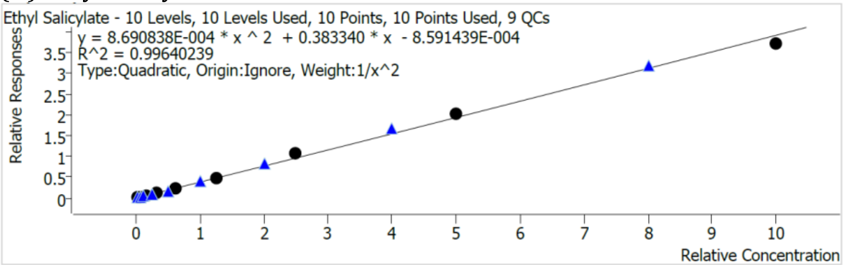


(O) trans-Cinnamaldehyde

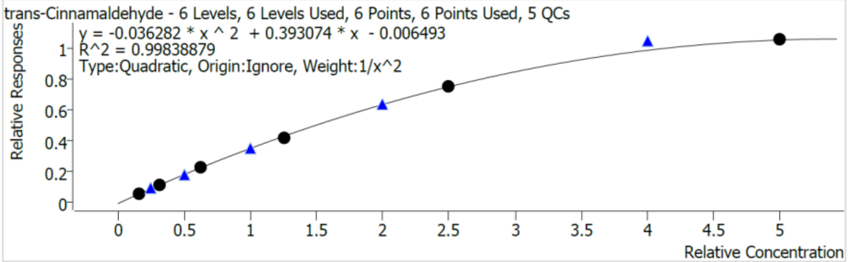


(P) Triacetin

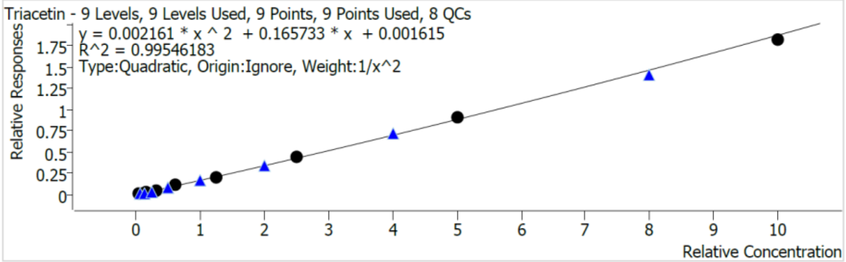

(Q) Eugenol

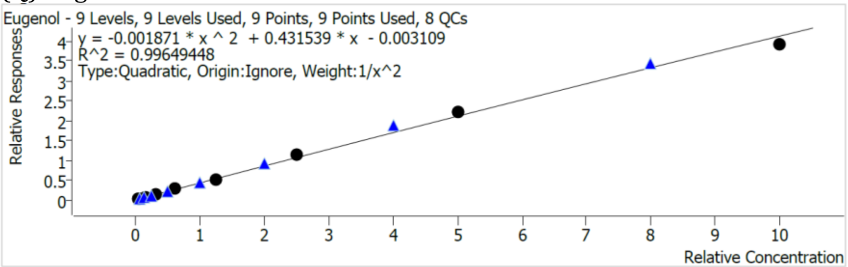


(R) Vanillin

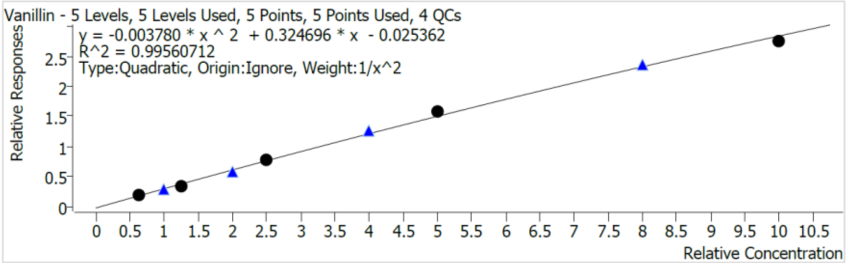


(S) Ethyl Vanillin

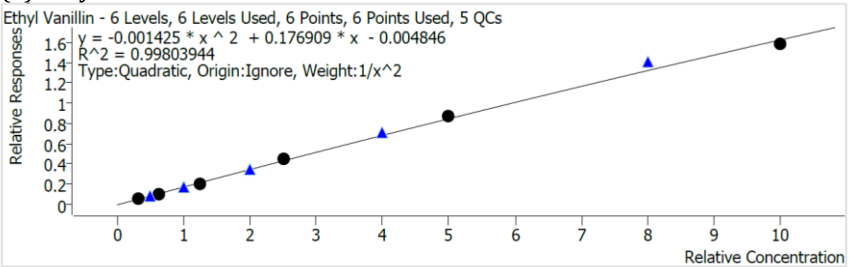


(T) Isovanillin

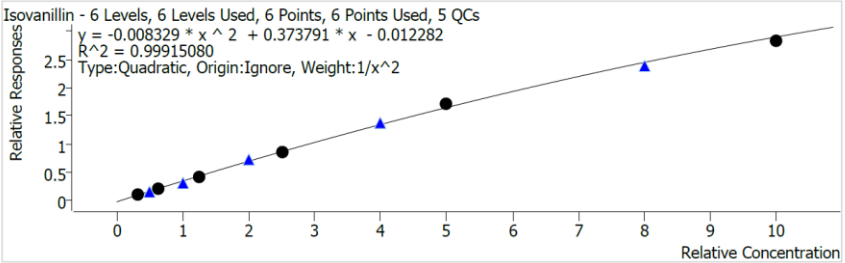


Circles (black) indicate calibration standards within the working calibration range (Supplemental Table 3) and are included in the calculation of the curve and corresponding $r^2$ value.
Triangles (blue) indicate quality control standards within the working calibration range.
